# Change of world-record rankings of shot put and hammer throw due to the effects of Earth rotation and athlete’s height

**DOI:** 10.1038/s41598-023-36665-5

**Published:** 2023-06-27

**Authors:** Gábor Horváth, Dénes Hegedűs, Judit Slíz-Balogh

**Affiliations:** Department of Biological Physics, ELTE Eötvös Loránd University, Pázmány Péter sétány 1, Budapest, 1117 Hungary

**Keywords:** Biophysics, Environmental sciences, Physics

## Abstract

The differences between the consecutive world-records of shot put and hammer throw tendentiously decrease. Therefore, nowadays it would be worth taking into account the influence of certain environmental factors on the range *L*, such as the latitude and release azimuth direction. Both factors exert influence on *L* by the centrifugal acceleration *a*_centrigugal_ and Coriolis acceleration *a*_Coriolis_ induced by the Earth’s rotation. The aim of this work is to reveal how the world-record ranking numbers would change, if *a*_centrigugal_ and *a*_Coriolis_ as well as the athletes’ height *h* were taken into account during the validation of the outdoor world records of senior female and male shot-putters and hammer-throwers. Using computer modelling, we reconstruct here the release velocities *v* of shots and hammers and the normalized muscle work of shot-putters achieved at the 20 best consecutive world records. We determined numerous changes of world-record ranking numbers of shot put and hammer throw due to the influence of *a*_centrigugal_, *a*_Coriolis_ and *h*. Height *h* has the largest effect on the range *L*, *a*_centrigugal_ has a medium influence, and *a*_Coriolis_ possesses the least impact on *L*. The physically most correct way would be to consider the release velocity *v* of the shot/hammer (easily measurable with an ultrasound/laser Doppler gauge) as the real performance of athletes, because it is practically independent of the environmental factors, and thus is a much better ranking measure of world records than the range.

## Introduction

Among the main olimpic throw sports – shot put, hammer throw, discus throw, javelin throw^1^ – in this work we deal only with the physics of the two most inertially dominated and least aerodynamically influenced throws, the shot put and the hammer throw. The differences between the ranges (throw distances) of the consecutive world records tendentiously decrease in both sports, because the performance of athletes approaches its upper limit^2^. Therefore, nowadays it would already be worth taking into account the influence of the following relevant environmental factors on the range *L*: wind speed, altitude, air pressure, air temperature, ground obliquity, latitude and azimuth direction of the throw. The latter two variables affect *L* through the centrifugal and Coriolis inertial accelerations induced by the Earth’s rotation.

The mechanics of the shot put and hammer throw have been thoroughly investigated with analytical calculations, computer simulations and experimental measurements^3–21^. Analysing the progression of the world records of shot put and hammer throw, Mizera and Horváth^22^ have demonstrated that the time has come to take into account the effect of certain environmental variables on *L* of both throwing sports.

In an accelerating and rotating system of coordinates with time-dependent linear acceleration vector *a*_linear_(*t*) and angular velocity vector ω(*t*) – as the Earth, for example – the motion equation of a thrown implement (shot or hammer) with mass *m* is^23^:

1 $$\begin{gathered} \underline {\text{a}} = \underline {\text{F}}/m{-}\underline {\text{a}}_{{{\text{linear}}}} + \underline {\text{a}}_{{{\text{Euler}}}} + \underline {\text{a}}_{{{\text{Coriolis}}}} + \underline {\text{a}}_{{{\text{centrifugal}}}} , \hfill \\ \underline{a}_{{{\text{Euler}}}} = \underline{r} \times \underline{{{\dot{\omega }}}} , \hfill \underline {\text{a}}_{{{\text{Coriolis}}}} = { 2}\underline {\nu } \times \underline {\omega } , \underline {\text{a}}_{{{\text{centrifugal}}}} = \underline {\omega } \times (\underline{r} \times \underline {\omega } ), \hfill \\ \end{gathered}$$

where *F* is the net real external force, *a*_linear_ is the linear acceleration vector, $\underline{a}_{{{\text{Euler}}}} = \underline{r} \times \underline{{{\dot{\omega }}}}$ is the Euler’s inertial acceleration vector due to the angular acceleration $\underline{{{\dot{\omega }}}} = {\text{d}}\underline{{\upomega }} (t)/{\text{d}}t$, *a*_Coriolis_ = 2*v* × ω and *a*_centrifugal_ = ω × (*r* × ω) are the Coriolis and centrifugal inertial acceleration vectors, where *t* is time and × represents vector cross product.

Mizera and Horváth^22^, Horváth^2^, and Jánosi and Bántay^24^ have studied the decreasing/increasing effects of the centrifugal and Coriolis accelerations on shot put and hammer throw ranges. Surveying the astronomical and geophysical literature^25,26^, Pálfi^27^ studied the four inertial accelerations in (1). She and her supervisor (Gábor Hováth) estimated the magnitude of the inertial accelerations *a*_tidal,Earth-Moon_, *a*_tidal,Earth-Sun_, *a*_tidal,Earth-Galaxis_, *a*_Euler_(due to general precession), *a*_Euler_(due to lunisolar nutation), *a*_Euler_(due to nutation during planetary precession) and *a*_Euler_(due to deceleration of Earth’s rotation) on the Earth’s surface because of the tidal force of the Galaxis, Sun and Moon, as well as the temporal change of the angular vector ω(*t*) of the Earth. They found that these inertial accelerations are 10^–6^–10^–25^ times smaller than the Newtonian gravitational acceleration *g*_Newton_ = 9.832 m/s^2^ (at the Poles), therefore they can be neglected in computation of the motion of the shot and hammer. At the same time, since the inertial accelerations *a*_centrifugal_ and *a*_Coriolis_ are only 10^–3^ and 10^–4^ times smaller than *g*_Newton_, respectively, it is worth taking into account their influence on the shot put and hammer throw ranges. According to Mizera and Horváth^22^, Horváth^2^ and Jánosi and Bántay^24^, the acceleration *a*_centrifugal_ changes the ranges of the hammer throw/shot put by an order of dm/cm, while *a*_Coriolis_ causes changes only in order of cm/mm.

We hypothesized that the world-record rankings of shot put and hammer throw change if the effects of the athletes’ height *h*, as well as *a*_centrifugal_ and *a*_Coriolis_ on the range *L* are taken into account. Using computer modelling, we study here how the world-record ranking numbers of the outdoor senior female and male shot put and hammer throw would change, if *a*_centrifugal_, *a*_Coriolis_ and *h* were taken into account in the validation. A preliminary analytical investigation of this problem has been published by Hegedűs^28^.

The release height *H* is proportional to the athlete’s height *h*. Although *H* trivially influences the range *L* (*L* increases/decreases with increasing/decreasing *H* at given values of the release angle and velocity), this work is the first which proves that some world-record ranking numbers of shot put and hammer throw would change, if the effects of *h* as well as *a*_centrifugal_ and *a*_Coriolis_ were taken into account. We also reveal which of these three factors is the most and least responsible for these changes, and suggest how the environmental effects on *L* could be minimized or even eliminated.

## Materials and methods

### Ethics declarations

We confirm that for our theoretical and computational studies no institutional permission, licence, approval or consent were necessary. The used data of athletes (thrower’s name, nationality and height) originate from public sources available from the Internet and given in Tables 1, 2, 3 and 4 and Supplementary Tables S1–S4. We also confirm that all theoretical and computational methods were carried out in accordance with relevant guidelines and regulations. Finally, we confirm that no experiments were carried out with humans.

**Table 1:** Data of the 20 best consecutive world records of outdoor senior male shot-putters.

*i*	*L*_i_ (m)	φ_i_ (rad)	*g*_i_ (m/s^2^)	Thrower’s name (nationality)	*h*_i_ (m)	*H*_i_ (m)	*v*_i_ (m/s)	*A*_i_ (m^2^/s^2^)
1	23.37	0.7686	9.805	Ryan Crouser (USA)	2.01	2.320	*j* = 1. *v*_1_ = 14.559	*j* = 1. *A*_1_ = 235.581
2	23.12	0.5945	9.797	Randy Barnes (USA)	1.95	2.260	*j* = 2. *v*_2_ = 14.488	*j* = 2. *A*_2_ = 233.483
3	23.06	0.6198	9.798	Ulf Timmermann (GDR)	1.94	2.250	*j* = 3. *v*_3_ = 14.471	*j* = 3. *A*_3_ = 233.003
4	22.91	0.7657	9.805	Alessandro Andrei (ITA)	1.91	2.220	*j* = 4. *v*_4_ = 14.435	*j* = 4. *A*_4_ = 231.962
5	22.84	0.7657	9.805	Alessandro Andrei (ITA)	1.91	2.220	*j* = 5. *v*_5_ = 14.410	*j* = 5. *A*_5_ = 231.249
6	22.72	0.7657	9.805	Alessandro Andrei (ITA)	1.91	2.220	*j* = 6. *v*_6_ = 14.367	*j* = 6. *A*_6_ = 230.026
7	22.64	0.9166	9.813	Udo Beyer (GDR)	1.94	2.250	*j* = 7. *v*_7_ = 14.334	*j* = 7. *A*_7_ = 229.079
8	22.62	0.9166	9.813	Ulf Timmermann (GDR)	1.94	2.250	*j* = 8. *v*_8_ = 14.327	*j* = 8. *A*_8_ = 228.875
9	22.22	0.5943	9.797	Udo Beyer (GDR)	1.94	2.250	*j* = 9. *v*_9_ = 14.172	*j* = 9. *A*_9_ = 224.435
10	22.15	1.0072	9.817	Udo Beyer (GDR)	1.94	2.250	*j* = 10. *v*_10_ = 14.162	*j* = 10. *A*_10_ = 224.180
11	22.00	0.8538	9.810	Aleksandr Baryshnikov (URS)	1.98	2.290	*j* = 11. *v*_11_ = 14.087	*j* = 11. *A*_11_ = 222.072
**12**	**21.85**	**0.3721**	**9.787**	**Terence Albritton (USA)**	**1.94**	**2.250**	***j* = 13. *v***_**12**_** = 14.032**	***j* = 13. *A***_**12**_** = 220.450**
**13**	**21.82**	**0.6515**	**9.799**	**Allan Feuerbach (USA)**	**1.86**	**2.170**	***j* = 12. *v***_**13**_** = 14.060**	***j* = 12. *A***_**13**_** = 221.261**
14	21.78	0.5341	9.794	Randel Matson (USA)	2.01	2.320	*j* = 14. *v*_14_ = 13.985	*j* = 14. *A*_14_ = 219.163
15	21.52	0.5341	9.794	Randel Matson (USA)	2.01	2.320	*j* = 15. *v*_15_ = 13.891	*j* = 15. *A*_15_ = 216.526
16	20.68	0.5943	9.797	Dallas Long (USA)	1.93	2.240	*j* = 16. *v*_16_ = 13.613	*j* = 16. *A*_16_ = 208.906
17	20.20	0.5943	9.797	Dallas Long (USA)	1.93	2.240	*j* = 17. *v*_17_ = 13.433	*j* = 17. *A*_17_ = 204.041
18	20.10	0.5943	9.797	Dallas Long (USA)	1.93	2.240	*j* = 18. *v*_18_ = 13.396	*j* = 18. *A*_18_ = 203.029
**19**	**20.08**	**0.5943**	**9.797**	**Dallas Long (USA)**	**1.93**	**2.240**	***j* = 20. *v***_**19**_** = 13.388**	***j* = 20. *A***_**19**_** = 202.826**
**20**	**20.06**	**0.5938**	**9.797**	**Williem Nieder (USA)**	**1.90**	**2.210**	***j* = 19. *v***_**20**_** = 13.392**	***j* = 19. *A***_**20**_** = 202.934**

*i* ranking number, *L*_i_ world-record range, φ_i_ latitude of the throwing event, *g*_i_ local gravitational acceleration, *h*_i_ height of the athlete, *H*_i_ release height of the thrown shot, *v*_i_ computationally reconstructed release velocity of the shot, *A*_i_ normalized muscle work of shot-putters calculated from (11). In rows *i* = 12., 13., 19. and 20. with bold data, the ranking number *i* of range *L* differs from the sequential number by size *j* of *v* and *A* (*i* ≠ *j*). https://www.worldathletics.org/records/by-progression/15758.

**Table 2:** Data of the 20 best consecutive world records of outdoor senior female shot-putters.

*i*	*L*_i_ (m)	φ_i_ (rad)	*g*_i_ (m/s^2^)	Thrower’s name (nationality)	*h*_i_ (m)	*H*_i_ (m)	*v*_i_ (m/s)	*A*_i_ (m^2^/s^2^)
1	22.63	0.9730	9.816	Natalya Lisovskaya (URS)	1.88	2.189	*j* = 1. *v*_1_ = 14.391	*j* = 1. *A*_1_ = 230.743
2	22.60	0.9730	9.816	Natalya Lisovskaya (URS)	1.88	2.189	*j* = 2. *v*_2_ = 14.381	*j* = 2. *A*_2_ = 230.434
**3**	**22.53**	**0.7608**	**9.805**	**Natalya Lisovskaya (URS)**	**1.88**	**2.189**	***j* = 4. *v***_**3**_** = 14.348**	***j* = 4. *A***_**3**_** = 229.456**
**4**	**22.45**	**0.9144**	**9.813**	**Ilona Slupianek (GDR)**	**1.79**	**2.099**	***j* = 3. *v***_**4**_** = 14.359**	***j* = 3. *A***_**4**_** = 229.791**
5	22.36	0.8070	9.807	Ilona Slupianek (GDR)	1.79	2.099	*j* = 5. *v*_5_ = 14.322	*j* = 5. *A*_5_ = 228.725
6	22.32	0.8432	9.809	Helena Fibingerova (TCH)	1.79	2.099	*j* = 6. *v*_6_ = 14.309	*j* = 6. *A*_6_ = 228.359
7	21.99	0.8716	9.811	Helena Fibingerova (TCH)	1.79	2.099	*j* = 7. *v*_7_ = 14.191	*j* = 7. *A*_7_ = 225.010
8	21.89	0.7360	9.804	Ivanka Khristova (BUL)	1.72	2.029	*j* = 8. *v*_8_ = 14.177	*j* = 8. *A*_8_ = 224.578
9	21.87	0.7360	9.804	Ivanka Khristova (BUL)	1.72	2.029	*j* = 9. *v*_9_ = 14.169	*j* = 9. *A*_9_ = 224.372
10	21.67	0.8872	9.812	Marianne Adam (GDR)	1.83	2.139	*j* = 10. *v*_10_ = 14.060	*j* = 10. *A*_10_ = 221.312
**11**	**21.60**	**0.9166**	**9.813**	**Marianne Adam (GDR)**	**1.83**	**2.139**	***j* = 12. *v***_**11**_** = 14.035**	***j* = 12. *A***_**11**_** = 220.615**
**12**	**21.57**	**0.8592**	**9.810**	**Helena Fibingerova (TCH)**	**1.79**	**2.099**	***j* = 11. *v***_**12**_** = 14.038**	***j* = 11. *A***_**12**_** = 220.668**
13	21.45	0.7540	9.805	Nadezhda Chizhova (URS)	1.74	2.049	*j* = 13. *v*_13_ = 14.009	*j* = 13. *A*_13_ = 219.860
14	21.20	0.8699	9.811	Nadezhda Chizhova (URS)	1.74	2.049	*j* = 14. *v*_14_ = 13.921	*j* = 14. *A*_14_ = 217.425
15	21.03	0.8402	9.809	Nadezhda Chizhova (URS)	1.74	2.049	*j* = 15. *v*_15_ = 13.857	*j* = 15. *A*_15_ = 215.634
16	20.63	0.7608	9.805	Nadezhda Chizhova (URS)	1.74	2.049	*j* = 16. *v*_16_ = 13.705	*j* = 16. *A*_16_ = 211.440
17	20.43	0.9730	9.816	Nadezhda Chizhova (URS)	1.74	2.049	*j* = 17. *v*_17_ = 13.638	*j* = 17. *A*_17_ = 209.624
18	20.43	0.6629	9.800	Nadezhda Chizhova (URS)	1.74	2.049	*j* = 18. *v*_18_ = 13.627	*j* = 18. *A*_18_ = 209.282
19	20.10	0.6629	9.800	Nadezhda Chizhova (URS)	1.74	2.049	*j* = 19. *v*_19_ = 13.502	*j* = 19. *A*_19_ = 205.901
20	20.10	0.9166	9.813	Margitta Gummel (GDR)	1.77	2.079	*j* = 20. *v*_20_ = 13.499	*j* = 20. *A*_20_ = 205.856

*i* ranking number, *L*_i_ world-record range, φ_i_ latitude of the throwing event, *g*_i_ local gravitational acceleration, *h*_i_ height of the athlete, *H*_i_ release height of the thrown shot, *v*_i_ computationally reconstructed release velocity of the shot, *A*_i_ normalized muscle work of shot-putters calculated from (11). In rows *i* = 3., 4., 11. and 12. with bold data, the ranking number *i* of range *L* differs from the sequential number by size *j* of *v* and *A* (*i* ≠ *j*). https://www.worldathletics.org/records/by-progression/5543.

**Table 3:** Data of the 20 best consecutive world records of outdoor senior male hammer-throwers.

*i*	*L*_i_ (m)	φ_i_ (rad)	*g*_i_ (m/s^2^)	Thrower’s name (nationality)	*h*_i_ (m)	*H*_i_ (m)	*v*_i_ (m/s)
1	86.74	0.8514	9.810	Yuriy Sedykh (URS)	1.85	1.778	*j* = 1. *v*_1_ = 29.680
2	86.66	1.0374	9.819	Yuriy Sedykh (URS)	1.85	1.778	*j* = 2. *v*_2_ = 29.679
3	86.34	0.9058	9.812	Yuriy Sedykh (URS)	1.85	1.778	*j* = 3. *v*_3_ = 29.609
4	84.14	0.9730	9.816	Sergey Litvinov (URS)	1.80	1.728	*j* = 4. *v*_4_ = 29.216
5	83.98	0.9730	9.816	Sergey Litvinov (URS)	1.80	1.728	*j* = 5. *v*_5_ = 29.186
6	81.80	0.9730	9.816	Yuriy Sedykh (URS)	1.85	1.778	*j* = 6. *v*_6_ = 28.767
7	81.66	0.7608	9.805	Sergey Litvinov (URS)	1.80	1.728	*j* = 7. *v*_7_ = 28.734
8	80.64	0.7573	9.805	Yuriy Sedykh (URS)	1.85	1.778	*j* = 8. *v*_8_ = 28.531
9	80.46	0.7573	9.805	Jüri Tamm (URS)	1.91	1.838	*j* = 9. *v*_9_ = 28.486
10	80.38	0.7573	9.805	Yuriy Sedykh (URS)	1.85	1.778	*j* = 10. *v*_10_ = 28.482
**11**	**80.32**	**0.8496**	**9.810**	**Karl-Hans Riehm (FRG)**	**1.95**	**1.878**	***j* = 12. *v***_**11**_** = 28.460**
**12**	**80.14**	**0.9730**	**9.816**	**Boris Zaychuk (URS)**	**1.80**	**1.728**	***j* = 11. *v***_**12**_** = 28.461**
13	79.30	0.8746	9.811	Walter Schmidt (FRG)	1.92	1.848	*j* = 13. *v*_13_ = 28.272
14	78.50	0.8617	9.810	Karl-Hans Riehm (FRG)	1.95	1.878	*j* = 14. *v*_14_ = 28.111
15	77.56	0.8617	9.810	Karl-Hans Riehm (FRG)	1.95	1.878	*j* = 15. *v*_15_ = 27,930
**16**	**76.70**	**0.8617**	**9.810**	**Karl-Hans Riehm (FRG)**	**1.95**	**1.878**	***j* = 17. *v***_**16**_** = 27.763**
**17**	**76.66**	**0.8402**	**9.809**	**Aleksey Spiridonov (URS)**	**1.92**	**1.848**	***j* = 18. *v***_**17**_** = 27.759**
**18**	**76.60**	**0.8961**	**9.812**	**Reinhard Theimer (GDR)**	**1.84**	**1.768**	***j* = 16. *v***_**18**_** = 27.767**
19	76.40	0.8437	9.809	Walter Schmidt (FRG)	1.92	1.848	*j* = 19. *v*_19_ = 27.709
20	75.48	0.8835	9.811	Anatoliy Bondarchuk (URS)	1.83	1.758	*j* = 20. *v*_20_ = 27.549

*i* ranking number, *L*_i_ world-record range, φ_i_ latitude of the throwing event, *g*_i_ local gravitational acceleration, *h*_i_ height of the athlete, *H*_i_ release height of the thrown hammer, *v*_i_ computationally reconstructed release velocity of the hammer. In rows *i* = 11., 12., 16., 17. and 18. with bold data, the ranking number *i* of range *L* differs from the sequential number by size *j* of *v* (*i* ≠ *j*). https://www.worldathletics.org/records/by-progression/16118.

**Table 4:** Data of the 20 best consecutive world records of outdoor senior female hammer-throwers.

*i*	*L*_i_ (m)	φ_i_ (rad)	*g*_i_ (m/s^2^)	Thrower’s name (nationality)	*h*_i_ (m)	*H*_i_ (m)	*v*_i_ (m/s)
1	82.98	0.9116	9.813	Anita Wlodarczyk (POL)	1.78	1.708	*j* = 1. *v*_1_ = 29.642
2	82.29	− 0.3999	9.788	Anita Wlodarczyk (POL)	1.78	1.708	*j* = 2. *v*_2_ = 29.467
3	81.08	0.9563	9.815	Anita Wlodarczyk (POL)	1.78	1.708	*j* = 3. *v*_3_ = 29.264
4	79.58	0.9166	9.813	Anita Wlodarczyk (POL)	1.78	1.708	*j* = 4. *v*_4_ = 28.957
5	79.42	0.8985	9.812	Betty Heidler (GER)	1.75	1.678	*j* = 5. *v*_5_ = 28.929
6	78.30	0.9271	9.814	Anita Wlodarczyk (POL)	1.78	1.708	*j* = 6. *v*_6_ = 28.698
7	77.96	0.9166	9.813	Anita Wlodarczyk (POL)	1.78	1.708	*j* = 7. *v*_7_ = 28.627
8	77.80	1.0374	9.819	Tatyana Lysenko (RUS)	1.86	1.788	*j* = 8. *v*_8_ = 28.588
9	77.41	0.9704	9.816	Tatyana Lysenko (RUS)	1.86	1.788	*j* = 9. *v*_9_ = 28.504
10	77.26	0.9460	9.814	Gulfiya Khanafeyeva (RUS)	1.73	1.658	*j* = 10. *v*_10_ = 28.494
11	77.06	0.9730	9.816	Tatyana Lysenko (RUS)	1.86	1.788	*j* = 11. *v*_11_ = 28.432
12	76.07	0.8306	9.809	Mihaela Melinte (ROU)	1.70	1.628	*j* = 12. *v*_12_ = 28.248
13	76.05	0.8306	9.809	Mihaela Melinte (ROU)	1.70	1.628	*j* = 13. *v*_13_ = 28.243
14	75.97	0.7990	9.807	Mihaela Melinte (ROU)	1.70	1.628	*j* = 14. *v*_14_ = 28.224
15	75.29	0.7990	9.807	Mihaela Melinte (ROU)	1.70	1.628	*j* = 15. *v*_15_ = 28.083
16	73.14	0.7959	9.807	Mihaela Melinte (ROU)	1.70	1.628	*j* = 16. *v*_16_ = 27.635
17	73.10	0.8402	9.809	Olga Kuzenkova (RUS)	1.76	1.688	*j* = 17. *v*_17_ = 27.618
18	71.22	0.8402	9.809	Olga Kuzenkova (RUS)	1.76	1.688	*j* = 18. *v*_18_ = 27.222
19	69.58	0.7754	9.806	Mihaela Melinte (ROU)	1.70	1.628	*j* = 19. *v*_19_ = 26.880
20	69.42	0.8163	9.808	Mihaela Melinte (ROU)	1.70	1.628	*j* = 20. *v*_20_ = 26.849

*i* ranking number, *L*_i_ world-record range, φ_i_ latitude of the throwing event, *g*_i_ local gravitational acceleration, *h*_i_ height of the athlete, *H*_i_ release height of the thrown hammer, *v*_i_ computationally reconstructed release velocity of the hammer. In all 20 rows, the ranking number *i* of range *L* corresponds with the sequential number by size *j* of *v* (*i* = *j*). https://www.worldathletics.org/records/by-progression/5911.

### Athlete’s heights, release heights and release angles of shot put and hammer throw

In our model, some important variables – also influencing the performance – are kept constant, because their actual values during world record throws are unknown and cannot be reconstructed. These constant variables are the air temperature *T*, air pressure *p*, air density ρ, release angle α, wind speed *v*_wind_ and ground obliquity *GO*. Since *T* and *p* depends on the altitude *AL*, the density ρ depends on *T* and *p*, and the range *L* depends on ρ, the altitude *AL* also influences *L*. Because the actual *T*- and *p*-values in the world record throwing events are not known, we kept them as constant normal values at *AL* = 0 m.

The heights *h* of outdoor senior male and female shot-putters and hammer-throwers performing the 20 best consecutive world records originate from public internet sources (Supplemenatary Tables S1–S4). Only the height of the American shot-putter Terence Hillary Albritton was not available from such a public source. However, in March 1977 Tom Jordan has interviewed Albritton^29^. From this interview it turned out that the height of T. H. Albritton was *h* = 6 feets (= 1.822 m) + 4.5 inches (= 0.1143 m) = 1.94 m. Table 1 and Supplemenatary Table S1 contain this value.

**Figure 1:**
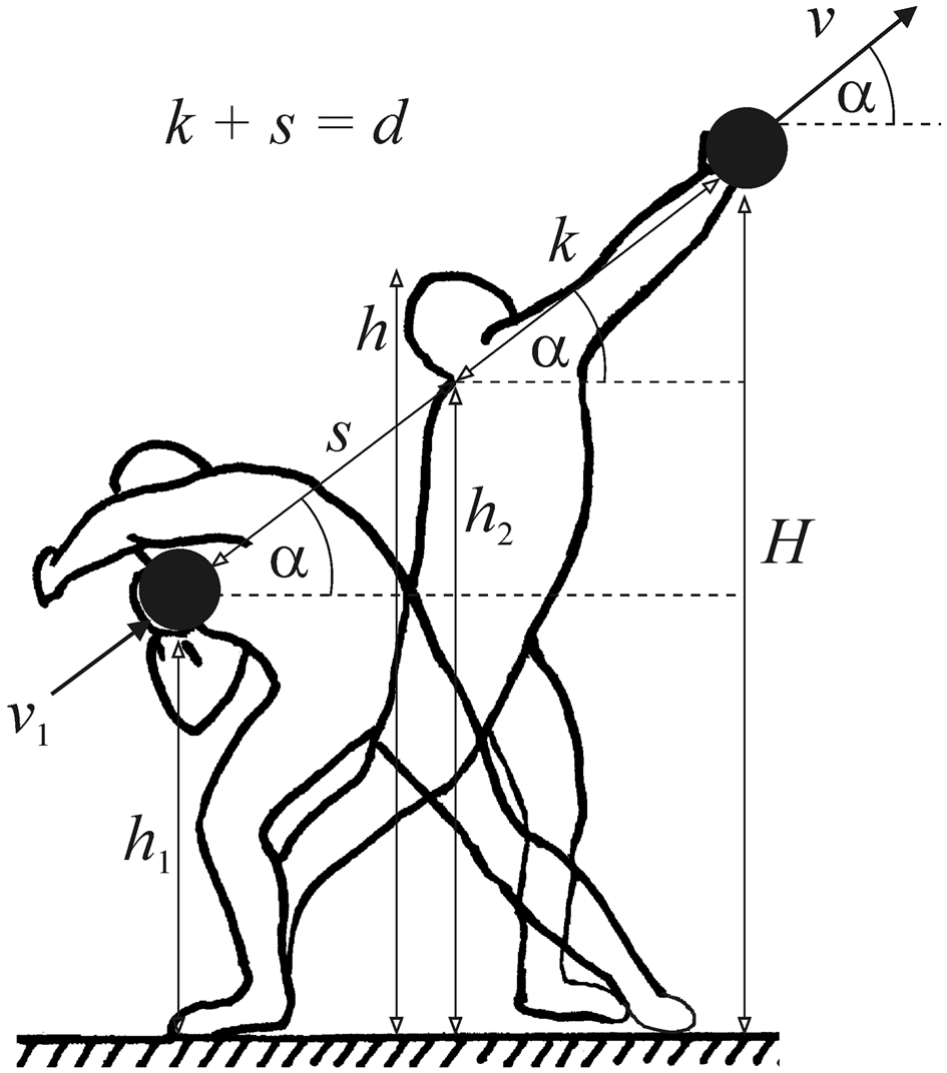
For calculation of the release height *H*, muscle work *W* and release velocity *v* of shot put. During the final acceleration of the shot, the shot-putter exerts work *W* along straight *d* = *k* + *s* against gravitation and to increase the kinetic energy of the shot.

**Figure 2:**
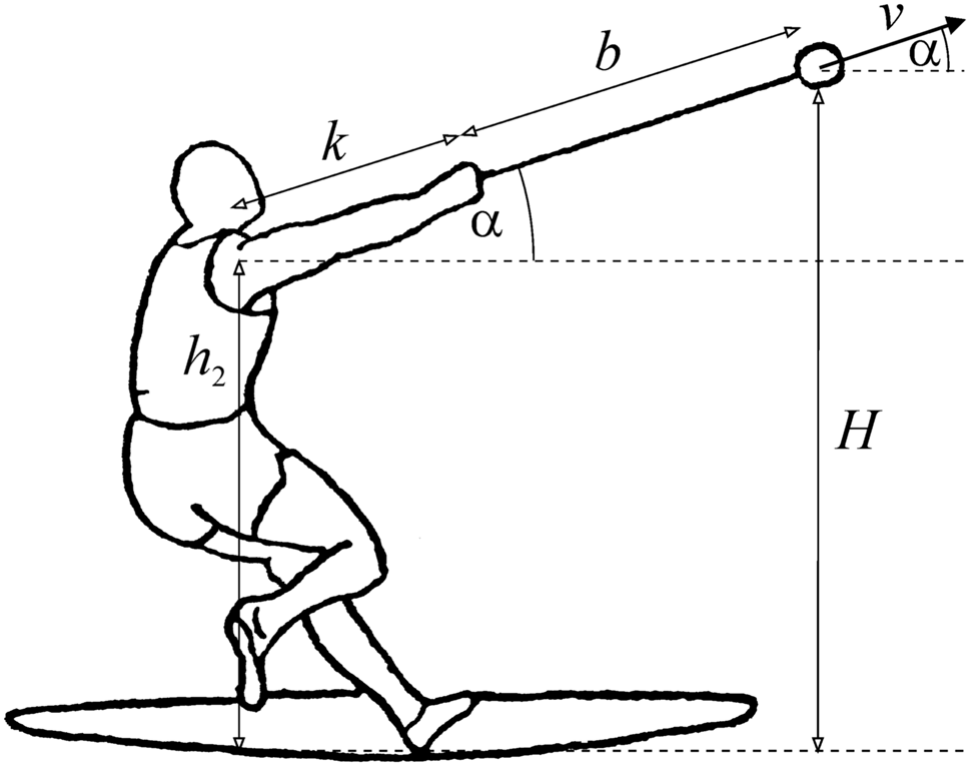
For calculation of the release height *H* of hammer throw.

According to Fig. 1, the release height *H* of shot put can be calculated as follows: *H* = *h*_2_ + *k*·sinα, where *h*_2_ is the shoulder height and *k* is the arm-sweep of the athlete, α is the release angle of the shot. Unfortunately, the individual values of *h*_2_ and *k* are unknown (not public), contrary to the body height *h*. Accepting the logical approximation that the length of the human body parts (in the present case *h*_2_ and *k*) is proportional to the height *h*, then knowing the individual *h* and average h* of shot-putters, *h*_2_ and *k* can be estimated in the following way: $h_{{2}} = h_{{2}}^{*} h/h^{*}$, $k = k^{*} h/h^{*}$, where $h_{{2}}^{*}$ and $k^{*}$ are the averages of the shoulder height and arm-sweep, respectively. Thus, the release height of shot put is: $H_{{{\text{shot}}}} = h_{{2}} + k \cdot \sin {\upalpha } = h_{{2}}^{*} h/h^{*} + (k^{*} h/h^{*} )\sin {\upalpha } = (h_{{2}}^{*} + k^{*} \sin {\alpha )}h/h^{*}$. On the basis of Fig. 2, we obtain similarly the release height of hammer throw: $H_{{{\text{hammer}}}} = h_{{2}} + (k + b)\sin {\upalpha } = h_{{2}}^{*} h/h^{*} + (k^{*} h/h^{*} + b)\sin {\upalpha }$, where *b* is the total length of the hammer and its handle. However, the problem is that it is practically impossible to get the average values of the shoulder height $h_{{2}}^{*}$ and arm-sweep $k^{*}$ of shot-putters and hammer-throwers. Therefore, the individual release heights *H* should be estimated in another way as follows: According to Megede and Hymans^30^, in case of the senior male shot put and hammer throw the average release heights measured from the ground of the shot/hammer are:

2 $$H^{*}_{{{\text{male}},{\text{shot}}}} = { 2}.{25}0{\text{ m}}, H^{*}_{{{\text{male}},{\text{hammer}}}} = { 1}.{8}00{\text{ m}}.$$

In case of the senior female shot put and hammer throw, there are only sporadic data for the average release heights H*_female,shot_ and H*_female,hammer_ of the shot/hammer, which few data have been measured only for a small number of female athletes^31,32^. Therefore, in view of the average heights h*_male,shot_, h*_male,hammer_, h*_female,shot_, h*_female,hammer_ of male and female shot-putters and hammer-throwers, we estimate the average release heights H*_female,shot_ and H*_female,hammer_ of female athletes in such a way that the difference h*_male_–h*_female_ between the average heights h*_male_ and h*_female_ of male and female athletes is extracted from the release height H*_male_ characteristic for males:

3 $$H^{*}_{{{\text{female}}}} = H^{*}_{{{\text{male}}}} {-} \, \left( {h^{*}_{{{\text{male}}}} {-}h^{*}_{{{\text{female}}}} } \right) \, = H^{*}_{{{\text{male}}}} {-}h^{*}_{{{\text{male}}}} + h^{*}_{{{\text{female}}}} .$$

Since the thrower-specific values of *H* are unknown and only the sport- and sex-dependent average release heights H* given by (2) and (3) are known/estimated, the thrower-specific height *H*_i_ can be estimated so that *H*_i_ is larger/smaller than H* by so much as the thrower-specific height *h*_i_ is larger/smaller than the sport- and sex-dependent average height h* of the athletes:

4 $$H_{{\text{i}}} \left( {h_{{\text{i}}} } \right) \, = H^{*} \, + h_{{\text{i}}} {-}h^{*}.$$

Using (3) and (4), we obtain the sport- and sex-specific release height of the shot/hammer:

5 $$\begin{gathered} H_{{{\text{i}},{\text{male}}}} \left( {h_{{{\text{i}},{\text{male}}}} } \right) \, \,\,\,\,\,\, = H^{*}_{{{\text{male}}}} + h_{{{\text{i}},{\text{male}}}} {-}h^{*}_{{{\text{male}}}} , \hfill \\ H_{{{\text{i}},{\text{female}}}} \left( {h_{{{\text{i}},{\text{female}}}} } \right) \, = \, \left( {H^{*}_{{{\text{male}}}} {-}h^{*}_{{{\text{male}}}} + h^{*}_{{{\text{female}}}} } \right) \, + h_{{{\text{i}},{\text{female}}}} {-}h^{*}_{{{\text{female}}}} , \hfill \\ \,\,\,\,\,\,\,\,\,\,\,\,\,\,\,\,\,\,\,\,\,\,\,\,\,\,\,\,\,\,\,\,\,\,\,\, = H^{*}_{{{\text{male}}}} + h_{{{\text{i}},{\text{female}}}} {-}h^{*}_{{{\text{male}}}} . \hfill \\ \end{gathered}$$

On the basis of (3) and Tables 1, 2, 3 and 4, the average heights for the 20 best consecutive world records of the male and female shot put are:

6 $$\begin{gathered} H^{*}_{{{\text{male}},{\text{shot}}}} = { 2}.{25}0{\text{ m}},\,\,\,\,\,\,\,\,\,\,H^{*}_{{{\text{male}},{\text{hammer}}}} = { 1}.{8}00{\text{ m}}, \hfill \\ h^{*}_{{{\text{male}},{\text{shot}}}} = { 1}.{\text{941 m}},\,\,\,\,\,\,\,\,\,\,\,\,h^{*}_{{{\text{female}},{\text{shot}}}} = { 1}.{\text{782 m}}, \hfill \\ h^{*}_{{{\text{male}},{\text{hammer}}}} = { 1}.{\text{872 m}},\,\,\,\,\,\,\,h^{*}_{{{\text{female}},{\text{hammer}}}} = { 1}.{\text{758 m}}, \hfill \\ H^{*}_{{{\text{female}},{\text{shot}}}} = { 2}.0{\text{91 m}},\,\,\,\,\,\,\,H^{*}_{{{\text{female}},{\text{hammer}}}} = { 1}.{\text{686 m}}. \hfill \\ \end{gathered}$$

Since the individual *k* and average k* of the athletes’ arm-sweeps are unknown, in the estimation of the release height *H* by (3), (4) and (5) we had to assume that *k* is independent of the athlete’s height *h*. However, with this assumption we committed only a negligible error, because the small (a few cm) individual differences of *k* contribute to the release height *H*_shot_ = *h*_2_ + *k*sinα and *H*_hammer_ = *h*_2_ + (*k* + *b*)sinα by much lesser extent – at shot put by *k*sinα and at hammer throw by (*k* + *b*)sinα – than the much larger shoulder height* h*_2_ (Figs. 1 and 2).

The longest ranges of shot put and hammer throw are ensured by the following ballistically optimal release angles measured from the horizontal^2,30,31^:

7 $$\alpha_{{{\text{shot}}}} = { 37}^{^\circ } , \alpha_{{{\text{hammer}}}} = { 44}^{^\circ } .$$

Although athletes always try to keep these α-values, there are often more or less deviations from them during throwing, because athletes fluctuate in their release angle from attempt to attempt, from athlete to athlete, and from technique to technique. Since the concrete α-values of the 80 world record throws studied by us are unknown and cannot be find out, in our model α is necessarily kept the constant values given in (7). However, this is not a problem at all, because our aim is to investigate how the athlete’s body height *h* as well as the Coriolis and centrifugal accelerations influence the world record ranking orders, rather than to study the effect of release angle α on range.

### International gravity formula on the Earth’s surface

Due to the centrifugal acceleration, the rotating Earth has a geoid shape, that can be well approximated by a rotational symmetric ellipsoid differing only slightly from a sphere. In average, the Earth’s surface bends in such a way that in every point it is perpendicular to the local gravitational acceleration vector. The latter has two components: the Newtonian gravitational acceleration and the centrifugal acceleration. Since the centrifugal acceleration increases with distance from the Earth’s rotation axis, its magnitude depends on the latitude. The magnitude of the Newtonian gravitational acceleration also changes with latitude, because the distance of the Earth’s surface from the Earth’s center depends on the latitude due to the geoid shape. On the Earth’s surface, the magnitude *g* of the gravitational acceleration is described with a high precision by the International Gravity Formula, called also Cassini’s formula as a function of the latitude φ^33,34^:

8 $$g\left( \varphi \right) \, = { 9}.{78}0{327}\cdot[{1 } + \, 0.00{53}0{24}\cdot{\text{sin}}^{{2}} \varphi \, {-} \, 0.00000{58}\cdot{\text{sin}}^{{2}} \left( {{2}\varphi } \right)] {\text{m}}/{\text{s}}^{{2}} ,$$

involving both the geoid shape (latitude-dependent Earth’s radius) and the centrifugal acceleration induced by the Earth’s rotation (latitude-dependent vertical component of the centrifugal acceleration).

### Normalized muscle work of shot-putters

In case of shot put, the release velocity *v* of the shot is^2^:

9 $$v = \sqrt {A - 2gd \cdot \sin {\upalpha }} ,$$

10 $$A = v_{1}^{2} + \frac{2W}{m},$$

where *m* is the shot’s mass, α is the release angle relative to the horizontal, *g* is the gravitational acceleration and *d* is the length of the straight along which the shot-putter accelerates the shot from velocity *v*_1_ to *v* (Fig. 1) with work *W*. From (9) we obtain:

11 $$A = v^{2} + 2gd \cdot \sin {\upalpha }{.}$$

Since the person-dependent value of *d* is not known for the different shot-putters, first we calculated with *d* = 1.7, 1.8, 1.9, 2.0, 2.1, 2.2, 2.3 m. However, the obtained changes of world-record rankings of shot put based on *A* were the same, being independent of *d* in the interval 1.7 m ≤ *d* ≤ 2.3 m^28^. Therefore, further on in this work we use *d* = 2 m.

According to (10), the quantity *A* given by (11) is essentially the physical measure of the shot-putter’s muscle work having two components: (1) $v_{1}^{2}$ = two times of the shot’s kinetic energy $mv_{1}^{2} /2$ per shot’s mass *m* exerted by the athlete in the nearly horizontal first section of acceleration. (2) 2*W*/*m* = two times of the muscle work *W* per mass *m* exerted by the shot-putter along the final tilted straight *d* of acceleration. Further on in this work, the quantity *A* is simply called as ’normalized muscle work’ instead of the exact but too long term ’twice the muscle work per shot’s mass’. Since *A* is practically independent of the environmental impacts, strictly speaking it should be considered as the real performance of shot-putters, rather than the range *L* that more or less depends on these impacts. Since on the basis of (9) the release velocity *v*[*g*(φ)] also depends on the gravitational acceleration *g*(φ) – being one of the most important environmental factors – the normalized muscle work *A* – being independent of *g* according to (10) – characterizes the real performance of shot-putters more correctly than *v*[*g*(φ)]. The consequence of this is that in case of the world-record shot puts, it would be pertinent to determine/reconstruct the normalized muscle work *A*^35^ and to reconsider/revise the ranking on the basis of the sequential number by size of *A*.

### Computer modelling of the motion of the shot and hammer

**Figure 3:**
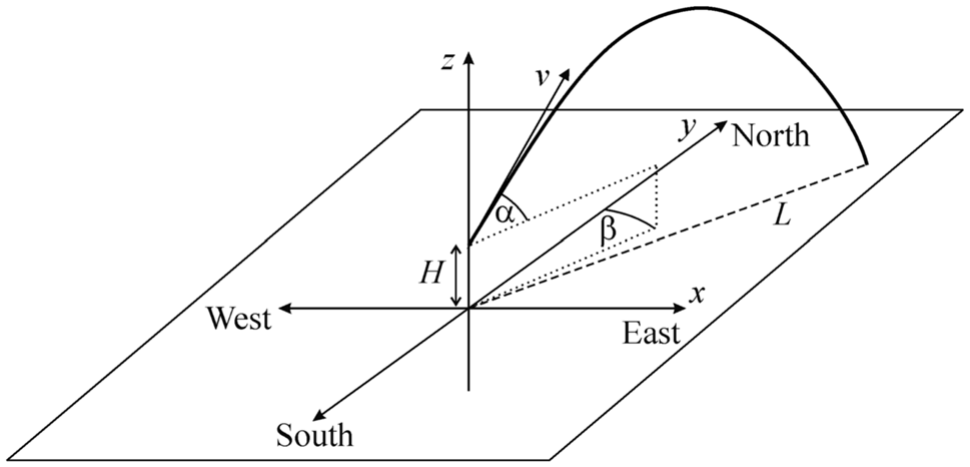
System of coordinates and variables of computer modelling of the motion of a shot/hammer.

We studied the motion of an implement (shot or hammer) in the Cartesian system of coordinates *x*-*y*-*z* fixed to the Earth’s surface (Fig. 3), where axes *x* and *y* are on the Earth’s surface, axis *z* is perpedicular to this surface, *x* points toward the geographical east, *y* toward north and *z* to the zenith. An athlete throws the implement from release height *H* from the ground, at release azimuth angle β clockwise from north and at release angle α relative to the horizontal. In this coordinate system, the general motion equation of a shot/hammer thrown with release velocity vector

12 $$\underline {\text{v}} = \, \left( {v\cdot{\text{cos}}\alpha \cdot{\text{sin}}\beta ,v\cdot{\text{cos}}\alpha \cdot{\text{cos}}\beta ,v\cdot{\text{sin}}\alpha } \right),$$

is the following:

13 $$\begin{gathered} m\frac{{{\text{d}}^{{2}} \underline{r} (t)}}{{{\text{d}}t^{2} }} = m \cdot \underline{g} (\phi ) + 2m\frac{{{\text{d}}\underline{r} (t)}}{{{\text{d}}t}} \times \underline{{\upomega }} - \frac{{kQ \cdot {\uprho }(p,T)}}{2} \cdot \left( {\frac{{{\text{d}}\underline{r} (t)}}{{{\text{d}}t}} - \underline{\upsilon }_{{{\text{wind}}}} } \right)^{2} \cdot \underline{n} , \hfill \\ \underline{n} = \frac{{\frac{{{\text{d}}\underline{r} (t)}}{{{\text{d}}t}} - \underline{v}_{{{\text{wind}}}} }}{{\left| {\frac{{{\text{d}}\underline{r} (t)}}{{{\text{d}}t}} - \underline{v}_{{{\text{wind}}}} } \right|}}, \hfill \\ \end{gathered}$$

where the meaning of variables and components are:*t*: time measured from the moment of release,*m*: mass of the shot/hammer. The shot’s/hammer’s mass of male throwers is *m*_male_ = 7.26 kg, while that of female ones is *m*_female_ = 4.00 kg, furthermore depending on the density, the diameter of the shot of males is 5.5–6.5 cm, and that of females is 4.75–5.5 cm^31,32^,φ: angle of the geographical latitude measured from the Equator. φ_max_ =  + 67.5° (Sodankylä) is the highest and φ_min_ = − 17.5° (Papeete) is the lowest latitude at which throwing competitions were ever organized^22^,β: release azimuth angle (direction) measured clockwise from north (β = 0°: north, β =  + 90°: east, β =  + 180°: south, β =  + 270° or − 90°: west),*p*: air pressure,

*T*: air temperature,ω: angular velocity vector of the Earth with magnitude ω = 2π/86 400/s = 7.27⋅10^−5^/s, parallel to the Earth’s rotation axis, pointing to the geographical North Pole with the following components^22^:

14 $$\begin{gathered} {\text{w}}_{{\text{x}}} = \, 0, \hfill \\ {\text{w}}_{{\text{y}}} = {\text{w}}\cdot{\text{cos}}\theta , \hfill \\ {\text{w}}_{{\text{z}}} = {\text{w}}\cdot{\text{sin}}\theta , \hfill \\ {\uptheta } = \arctan \left( {\frac{{R_{{\text{e}}}^{{2}} }}{{R_{{\text{p}}}^{{2}} }}\tan \phi } \right), \hfill \\ \end{gathered}$$

where θ is the angle of ω relative to the Earth’s surface at latitude angle φ, *R*_e_ = 6 378 000 m and *R*_p_ = 6 357 000 m are the average equatorial and polar Earth’s radii, respectively.*g*(φ): magnitude of the gravitational acceleration at latitude angle φ decribed by the Cassini’s formula (8) with the following components: *g*_x_ = 0, *g*_y_ = 0, *g*_z_ = − *g*(φ), including also the centrifugal acceleration induced by the Earth’s rotation. The change of *g* with the altitude is neglected in this work, because the majority of the Summer Olympic Games venue in the last 100 years has occurred at altitude *AL* < 100 m with only three exceptions: Munich (320 m), Atlanta (450 m) and Mexico City (2200 m)^22^.

$2\frac{{{\text{d}}\underline{r} }}{{{\text{d}}t}} \times \underline{\omega }$: Coriolis acceleration.*v*_wind_: velocity vector of wind. In this work *v*_wind_ = 0 is assumed (corresponding to a windless situation), because it is impossible to find out the magnitude and direction of *v*_wind_ in a given throwing event. Mizera and Horváth^22^ studied in detail the influence of wind on the range.

$\frac{{k{\uprho }Q}}{2} \cdot \left( {\frac{{{\text{d}}\underline{r} }}{{{\text{d}}t}} - \underline{v}_{{{\text{wind}}}} } \right)^{2}$: magnitude of the air drag due to air resistance, where *Q* is the projected area of the shot (*Q*_shot_ = 0.0095 m^2^, with radius *r*_shot_ = 5.5 cm) and hammer (*Q*_hammer_ = 0.0138 m^2^), *k* is the drag coefficient of the shot (*k*_shot_ = 0.47) and hammer (*k*_hammer_ = 0.7). The exact value of *k*_hammer_ varies along the flight trajectory and depends on the attitude of the wire and handle relative to the flight path. The handle performs a precession around the head during flight, the consequence of which is that its drag varies too. *k*_hammer_ = 0.7 is an averaged effective value^9^. In case of the typical average velocities of 15 and 30 m/s of the shot and hammer during flight, respectively^9^, the air drag is proportional to the square of velocity.ρ: air density depending on the air pressure *p* and air temperature *T* according to the equation of state of ideal gases:

15 $$\rho \, = p/\left( {BT} \right), B = { 287},0{\text{5 J}}/\left( {{\text{kgK}}} \right).$$

In our simulations, the standard/normal values of environmental parameters were the following: air temperature *T* = 293 K, air pressure *p* = 101 325 Pa, air density ρ = 1.21 kg/m^3^, ground obliquity *GO* = 0, and altitude *AL* = 0 m was assumed, because the influence of *AL* on the range is negligible for *AL* < 100 m^22^. Motion Eq. (13) involves all relevant environmental factors determining the flight trajectory of a thrown shot/hammer. We solved it with a Runge–Kutta numerical integrator of 4th order.

## Results

### Reconstruction of the release velocity of shots/hammers and the normalized muscle work of shot-putters at world records

Solving the motion Eq. (13) of implements (shots/hammers), we computationally reconstructed their release velocity *v* as follows: We took a given world-record range *L*. Assuming an initial release velocity of 30 m/s and solving (13) for the actual parameters (local gravitational acceleration *g*, release height *H*, release angle α, release azimuth angle β), we obtained the motion trajectory of the implement hitting the horizontal ground surface at a range *R*. If *R* was smaller or larger than *L*, then *v* was increased or decreased with Δ*v* = 10^–6^ m/s. After solving (13) with the use of the new value *v* + Δ*v* or *v*–Δ*v*, a new *R*-value was obtained. This iteration was performed until the difference |*R*–*L*| became smaller than ε = 10^–6^ m. Thus, the value of the release velocity *v* was reconstructed with an accuracy of Δ*v* = 10^–6^ m/s for a given world-record range *L*.

Tables 1, 2, 3 and 4 contain the relevant data of the 20 best consecutive world records of outdoor senior male and female shot-putters and hammer-throwers, such as the ranking number *i*, world-record range *L*_i_, latitude φ_i_ of the throwing event, local gravitational acceleration *g*_i_ calculated from the Cassini’s formula (8), name, nationality and height *h*_i_ of the throwers, thrower-specific release height *H*_i_ of the implement, computationally reconstructed release velocity *v*_i_ of the implement, and normalized muscle work *A*_i_ of shot-putters calculated from (11) for α_shot_ = 37°, α_hammer_ = 44°, *d* = 2.0 m and northern release azimuth angle β_N_ = 0°. The sequential numbers in columns of *v*_i_ and *A*_i_ reflect the sequential numbers by size of both variables. In the rows with bold data of Tables 1, 2, 3 and 4, the ranking number *i* of range *L* differs from the sequential number by size *j* of *v* and *A* (*i* ≠ *j*).

### Change of typical world record ranges versus azimuth, latitude and release height

In order to demonstrate the absolute changes in range *L* when the azimuth angle β, or the latitude angle φ, or the release height *H* varies, we performed the following analyses: A fixed computationally reconstructed release velocity *v* was taken, then β (0° ≤ β ≤ 360°), or φ (− 90° ≤ φ ≤  + 90°), or *H* (200 cm ≤ *H*_shot put_ ≤ 240 cm, 160 cm ≤ *H*_hammer throw_ ≤ 200 cm) was systematically varied from their minimum to maximum values, and *L* was computed. These computations give a clear indication of the magnitude of possible influences on *L* from the variability of β, φ and *H* for normal environmental conditions (*T*_air_ = 293 K, *p*_air_ = 101 325 Pa, ρ_air_ = 1.21 kg/m^3^, ground obliquity *GO* = 0, altitude *AL* = 0 m). We studied the following three cases:

(i) When β changed from 0° to 360°, then φ and *H* were the specific latitude and release height of a given world record.
(ii) When φ changed from − 90° to + 90°, then a northern azimuth angle β = 0° was assumed with the specific *H* of a given world record, because the azimuth angle is not known for the different world records.
(iii) When *H* changed from *H*_min_ to *H*_max_, then β = 0° was assumed (because the azimuth angles of world records are unknown) and φ was the specific latitude of a given world record.

**Figure 4:**
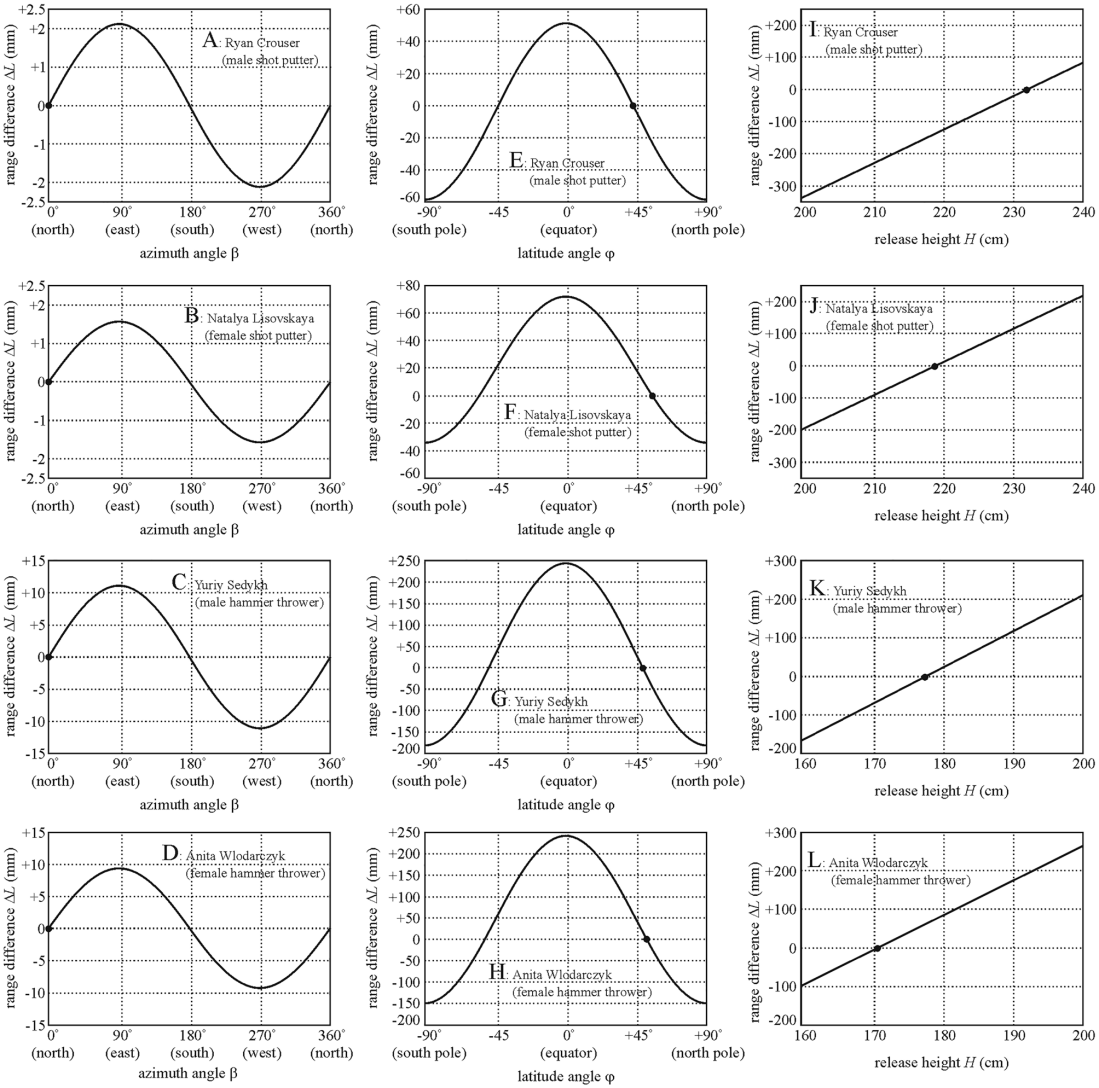
Range differences Δ*L*(β) = *L*(β)—*L*_world record_, Δ*L*(φ) = *L*(φ)—*L*_world record_ and Δ*L*(*H*) = *L*(*H*)–*L*_world record_ as functions of the azimuth angle β, latitude angle φ and release height *H* computed under normal environmental conditions (*T*_air_ = 293 K, *p*_air_ = 101 325 Pa, ρ_air_ = 1.21 kg/m^3^, ground obliquity *GO* = 0, altitude *AL* = 0 m) for the throws of Ryan Crouser (male shot-putter, *L*_world record_ = 23.37 m), Natalya Lisovskaya (female shot-putter, *L*_world record_ = 22.63 m), Yuriy Sedykh (male hammer-thrower, *L*_world record_ = 86.74 m) and Anita Wlodarczyk (female hammer-thrower, *L*_world record_ = 82.98 m), who are the recent world recorders. Their record point is marked by a dot in each subfigure. The numerical values of parameters characterizing these world records are given in Table 5.

Figure 4 shows the range differences Δ*L*(β) = *L*(β)–*L*_world record_, Δ*L*(φ) = *L*(φ)–*L*_world record_ and Δ*L*(*H*) = *L*(*H*)–*L*_world record_ versus β, φ and *H* computed for the world record throws of Ryan Crouser (male shot-putter, *L*_world record_ = 23.37 m), Natalya Lisovskaya (female shot-putter, *L*_world record_ = 22.63 m), Yuriy Sedykh (male hammer-thrower, *L*_world record_ = 86.74 m) and Anita Wlodarczyk (female hammer-thrower, *L*_world record_ = 82.98 m). The numerical values of parameters characterizing these world records are given in Table 5. Δ*L*(β) is a sinusoid function, Δ*L*(φ) is a parabola, while Δ*L*(*H*) is an increasing straight line. In Fig. 4 one can see the followings:

**Table 5:** Numerical values of parameters characterizing four recent world records of the shot put and hammer throw.

Thrower’s name	*L*_world record_ (m)	φ (rad, °)	*g* (m/s^2^)	*H* (m)	*v* (m/s)
Ryan Crouser (male shot-putter)	23.37	+ 0.7686 rad, + 44.04°	9.805	2.320	14.559
Natalya Lisovskaya (female shot-putter)	22.63	+ 0.9730 rad, + 55.75°	9.816	2.189	14.391
Yuriy Sedykh (male hammer-thrower)	86.74	+ 0.8514 rad, + 48.78°	9.810	1.778	29.680
Anita Wlodarczyk (female hammer-thrower)	82.98	+ 0.9116 rad, + 52.23°	9.813	1.708	29.642

*L*_world record_ world record range, φ latitude angle, *g* gravitational acceleration, *H* release height, *v* release velocity.

The change of β has the smallest influence on *L* because of the very small Coriolis acceleration relative to the centrifugal and gravitational accelerations. If β changes from 0° (north) through 90° (east), 180° (south) and 270° (west) to 360° (north), then *L*_world record_ = 23.37 m of Ryan Crouser would change between − 2.2 mm ≤ Δ*L*(β) ≤  + 2.2 mm (Fig. 4A), and for *L*_world record_ = 22.63 m of Natalya Lisovskaya − 1.6 mm ≤ Δ*L*(β) ≤  + 1.6 mm (Fig. 4B), for *L*_world record_ = 86.74 m of Yuriy Sedykh − 12 mm ≤ Δ*L*(β) ≤  + 12 mm (Fig. 4C), for *L*_world record_ = 82.98 m of Anita Wlodarczyk − 9 mm ≤ Δ*L*(β) ≤  + 9 mm (Fig. 4D).

The change of φ has a medium large influence on *L*, because the centrifugal acceleration is between the Coriolis and gravitational accelerations. If φ varies from − 90° (South Pole) through 0° (Equator) to + 90° (North Pole), then *L*_world record_ = 23.37 m of Ryan Crouser would change between − 60 mm ≤ Δ*L*(φ) ≤  + 50 mm (Fig. 4E), and for *L*_world record_ = 22.63 m of Natalya Lisovskaya − 32 mm ≤ Δ*L*(φ) ≤  + 77 mm (Fig. 4F), for *L*_world record_ = 86.74 m of Yuriy Sedykh − 180 mm ≤ Δ*L*(φ) ≤  + 250 mm (Fig. 4G), for *L*_world record_ = 82.98 m of Anita Wlodarczyk − 150 mm ≤ Δ*L*(φ) ≤  + 247 mm (Fig. 4H).

The variation of *H* has the largest influence on *L*, because the gravitational acceleration is much larger than the centrifugal and Coriolis accelerations. If in case of the shot put *H* varies from 200 to 240 cm, then *L*_world record_ = 23.37 m of Ryan Crouser would change between − 340 mm ≤ Δ*L*(*H*) ≤  + 80 mm (Fig. 4I), and for *L*_world record_ = 22.63 m of Natalya Lisovskaya − 200 mm ≤ Δ*L*(*H*) ≤  + 215 mm (Fig. 4J). If in case of the hammer throw *H* varies from 160 to 200 cm, then for *L*_world record_ = 86.74 m of Yuriy Sedykh − 170 mm ≤ Δ*L*(*H*) ≤  + 210 mm (Fig. 4K), and for *L*_world record_ = 82.98 m of Anita Wlodarczyk − 100 mm ≤ Δ*L*(*H*) ≤  + 275 mm (Fig. 4L).

### Changes of world-record rankings of shot put and hammer throw

The influence of the more or less differing environmental conditions in the different sites of throwing events on the range could be eliminated only, if the athletes threw in the same circumambiency. For example, when the throws would happen at the same gravitational acceleration *g*, toward the same release azimuth direction β and under the same meteorological conditions. Let us consider the imaginary situation that the athletes throw at the same *g* and β with the release velocities *v* performed at their world records, rather than at different *g* of different latitudes and toward different β. Tables 6 and 7 compare the ranges *L* in this way.

**Table 6:** Ranges *L*_i,j_ (in meter) (row: *i* = 19, 20; column: *j* = 1, 2, …, 6) of senior outdoor female shot-putters obtained with computer modelling for the computationally reconstructed release velocities *v*_i_ (m/s) in Table 2, gravitational acceleration *g*_j_ (m/s^2^) at latitude φ_j_ of throwing events, release angle α = 37° and release azimuth angles β_N_ = 0° (north), β_E_ = 90° (east), β_W_ = 270° (west).

*H*_i_ (m)	*v*_i_ (m/s)	*g*_1(1,2,17)_ = 9,816	*g*_2(3,8,9,13,16)_ = 9,805	*g*_3(4,10,11,20)_ = 9,813	*g*_4(5)_ = 9,807	*g*_5(6,7,12,14,15)_ = 9,809	*g*_6(18,19)_ = 9,800
		φ_1_ = 0.9730	φ_2_ = 0.7608	φ_3_ = 0.9144	φ_4_ = 0.8070	φ_5_ = 0.8432	φ_6_ = 0.6629
*i* = 19, *H*_19_ = 2.049	*i* = 19, *v*_19_ = 13.502	*k* = 20, N: 20.070, E: 20.072, W: 20.069	*k* = 20, N: 20.090, E: 20.092, W: 20.089	*k* = 20, N: 20.076, E: 20.077, W: 20.075	*k* = 20, N: 20.087, E: 20.088, W: 20.085	*k* = 20, N: 20.083, E: 20.085, W: 20.082	*i = 19., L*_*19*_* = 20.10*
							*k* = 20, N: 20.100, E: 20.101, W: 20.098
*i* = 20, *H*_20_ = 2.079	*i* = 20, *v*_20_ = 13.499	*k* = 19, N: 20.094, E: 20.095, W: 20.092	*k* = 19, N: 20.114, E: 20.115, W: 20.112	*i = 20., L*_*20*_* = 20.10*	*k* = 19, N: 20.110, E: 20.112, W: 20.108	*k* = 19, N: 20.106, E: 20.108, W: 20.105	*k* = 19, N: 20.123, E: 20.124, W: 20.121
				*k* = 19, N: 20.099, E: 20.100, W: 20.098			

The italic items *L*_*19*_*, L*_*20*_ and sequential numbers *i = 19., 20.* are equal with the 19. and 20. world records in Table 2. Ranges *L*_i_ in a given column *j* and the underlined sequential numbers *k* = 19., 20. represent those ranges and ranking numbers which could have been reached in a fictive throwing event at *g*_j_, as if both athletes had thrown at the same *g*_j_ with the release velocity *v*_i_ performed at the own world record with release angle α toward north (N: β_N_ = 0°), east (E: β_E_ = 90°) or west (W: β_W_ = 270°). Note that in all cases *i* differs from *k* (*i* ≠ *k*). The indices in parentheses of *g*_j_ are equal with the indices of the gravitational accelerations with practically the same magnitude (Δ*g*_max_ = 0,002 m/s^2^) in Table 2.

**Table 7:** Ranges *L*_i,j_ (in meter) (row: *i* = 1, 2, 10, 11; column: *j* = 1, 2, …, 5) of senior outdoor male hammer-throwers obtained with computer modelling for the computationally reconstructed release velocities *v*_i_ (m/s) in Table 3, gravitational acceleration *g*_j_ (m/s^2^) at latitude φ_j_ of the throwing events, release angle α = 44° and release azimuth angles β_N_ = 0° (north), β_E_ = 90° (east), β_W_ = 270° (west).

*H*_i_ (m)	*v*_i_ (m/s)	*g*_1(1,11,14,15,16,17,19,)_ = 9.810	*g*_2(2)_ = 9.819	*g*_3(3,13,18,20)_ = 9.812	*g*_4(4,5,6,12)_ = 9.816	*g*_5(7,8,9,10)_ = 9.805
		φ_1_ = 0.8514	φ_2_ = 1.0374	φ_3_ = 0.9058	φ_4_ = 0.9730	φ_5_ = 0.7608
*i* = 1, *H*_1_ = 1.778	*i* = 1, *v*_1_ = 29.680	*i = 1., L*_*1*_* = 86.74*	***k* = 1.(2.) N: 86.667**	***k* = 1.(2.) N: 86.725**	***k* = 1.(2.) N: 86.692**	***k* = 1.(2.) N: 86.782**
		***k* = 1.(2.) N: 86.741**				
		*k* = 1. E: 86.752	*k* = 1. E: 86.676	*k* = 1. E: 86.735	*k* = 1. E: 86.701	*k* = 1. E: 86.795
		***k* = 1.(2.) W: 86.730**	***k* = 1.(2.) W: 86.659**	***k* = 1.(2.) W: 86.714**	***k* = 1.(2.) W: 86.682**	***k* = 1.(2.) W: 86.770**
*i* = 2, *H*_2_ = 1.778	*i* = 2, *v*_2_ = 29.679	***k* = 2.(1.) N: 86.736**	*i = 2., L*_*2*_* = 86.66*	***k* = 2.(1.) N: 86.719**	***k* = 2.(1.) N: 86.687**	***k* = 2.(1.) N: 86.777**
			***k* = 2.(1.) N: 86.662**			
		***k* = 2.(1.) E: 86.747**	***k* = 2.(1.) E: 86.670**	***k* = 2.(1.) E: 86.730**	***k* = 2.(1.) E: 86.696**	***k* = 2.(1.) E: 86.789**
		*k* = 2. W: 86.725	*k* = 2. W: 86.653	*k* = 2. W: 86.709	*k* = 2. W: 86.677	*k* = 2. W: 86.765
*i* = 10, *H*_10_ = 1.778	*i* = 10, *v*_10_ = 28.482	*k* = 10, N: 80.343, E: 80.353, W: 80.333	*k* = 10, N: 80.274, E: 80.282, W: 80.267	*k* = 10, N: 80.327, E: 80.337, W: 80.318	*k* = 10, N: 80.297, E: 80.306, W: 80.289	*i = 10., L*_*10*_* = 80.38*
						*k* = 10. N: 80.381
						*k* = 10. E: 80.392
						***k* = 10.(11.) W: 80.370**
*i* = 11, *H*_11_ = 1.878	*i* = 11, *v*_11_ = 28.460	*i = 11., L*_*11*_* = 80.32*	*k* = 11, N: 80.253, E: 80.261, W: 80.246	*k* = 11, N: 80.306, E: 80.316, W: 80.297	*k* = 11, N: 80.276, E: 80.284, W: 80.268	*k* = 11. N: 80.360
		*k* = 11, N: 80.322, E: 80.332, W: 80.312				***k* = 11.(10.) E: 80.371**
						*k* = 11. W: 80.349

The italic items *L*_*1*_*, L*_*2*_*, L*_*10*_*, L*_*11*_ and sequential numbers *i = 1., 2., 10., 11.* are equal with the 1., 2., 10., 11. world records in Table 3. Ranges *L*_i_ in a given column *j* and the underlined sequential numbers *k* = 1., 2., 10., 11. represent those ranges and ranking numbers which could have been achieved in a fictive throwing event at gravitational acceleration *g*_j_, as if all four athletes had thrown at the same *g*_j_ with the release velocity *v*_i_ at the own world records with release angle α toward north (N: β_N_ = 0°), east (E: β_E_ = 90°) or west (W: β_W_ = 270°). The indices in parentheses of *g*_j_ are equal with the indices of the gravitational accelerations with practically the same magnitude (Δ*g*_max_ = 0.002 m/s^2^) in Table 3. In case of the bold data, *i* is not always equal with *k.*

According to Table 6, if the 19. (Nadezhda Chizhova) and the 20. (Margitta Gummel) shot-putters had thrown at the same gravitational acceleration *g* with the release velocities *v*_19_ = 13.502 m/s and *v*_20_ = 13.499 m/s achieved at their world records, then – independently of the release azimuth direction β and the actual *g* – their originally equal ranges *L*_19_ = *L*_20_ = 20.10 m would have changed in such a way that the range *L*_19,N,E,W_ of Nadezhda Chizhova would have been smaller than the range *L*_20,N,E,W_ of Margitta Gummel (*L*_19,N,E,W_ < *L*_20,N,E,W_). In this case their ranking numbers would have been interchanged with each other: 19. ↔ 20.

According to Table 2, Nadezhda Chizhova (*h*_19_ = 1.74 m) was lower by 3 cm than Margitta Gummel (*h*_20_ = 1.77 m), which decreased the range of the former. At the same time, Nadezhda Chizhova (*g*_19_ = 9.800 m/s^2^) had an advantage relative to Margitta Gummel (*g*_20_ = 9.813 m/s^2^) due to the smaller local gravitational acceleration *g*_19_ < *g*_20_, which increased the range of the former. The reason for the 19. ↔ 20. ranking number interchange occurring in case of shot puts at the same gravitational acceleration is the synergistic influence of *g* and *h* on the range. This interchange could not have been modified by the change of range because of the Coriolis acceleration due to the incidental difference of the release azimuth directions β.

If the world-record rankings were based on the release velocity *v* of the shot, then Nadezhda Chizhova (*v*_19_ = 13.502 m/s) would remain 19. and Margitta Gummel (*v*_20_ = 13.499 m/s) would keep her ranking number 20., because *v*_19_ > *v*_20_. However, in this case the ranking number interchanges 3. ↔ 4. and 11. ↔ 12. would occur due to the relations *v*_4_ = 14.359 m/s > *v*_3_ = 14.348 m/s and *v*_12_ = 14.038 m/s > *v*_11_ = 14.035 m/s (Table 2).

According to Table 7, in case of the 1. (Yuriy Sedykh) and the 2. (Yuriy Sedykh) hammer-throw world record, independently of the gravitational acceleration *g*, the 1. ↔ 2. ranking number interchange would have occurred, if both throws had happened at the same *g* with the release velocity *v* at the world record, and if the 1. throw had happened toward north (N) and the 2. throw toward east (E), when the relation *L*_1__,N_ < *L*_2,E_ would have been realized. (i) Then, the Coriolis acceleration would not have changed the range of the 1. northern (β_N_ = 0°) throw, while it would have slightly increased the range of the 2. eastward (β_E_ = 90°) throw. (ii) The larger release velocity *v*_1_ = 29.680 m/s of the 1. hammer throw increased the range relative to that of the 2. hammer throw with smaller *v*_2_ = 29.679 m/s. (iii) Since both world records have been achieved by the same hammer-thrower, Yuriy Sedykh, the height *h* did not play a role in this ranking number interchange. Hence, here the synergistic effects of the release azimuth direction β and release velocity *v* would have caused the 1. ↔ 2. ranking number interchange due to the relation *L*_1__,N_ < *L*_2,E_.

According to Table 7, the 1. ↔ 2. ranking number interchange would have occurred, if both throws had happened at the same *g* with the release velocity *v* at the world records, if the 1. throw had happened toward west (W) and the 2. throw toward north (N) or east (E), when the relation *L*_1__,W_ < *L*_2,N_ < *L*_2,E_ would have been realized. (i) In this case, the Coriolis acceleration would have decreased the range of the 1. westward (β_W_ = 270°) throw, while it would not have changed the range of the 2. northward (β_N_ = 0°) throw, furthermore it would have slightly increased the range of the 2. eastward (β_E_ = 90°) throw. (ii) The larger release velocity *v*_1_ = 29.680 m/s of the 1. hammer throw increased the range compared to that of the 2. throw with smaller *v*_2_ = 29.679 m/s. (iii) Since both world records have been performed by the same hammer-thrower, Yuriy Sedykh, the height *h* did not play a role in this ranking number interchange. Here again, the synergism of the release azimuth direction β and release velocity *v* would have caused the 1. ↔ 2. ranking number interchange because of the relation *L*_1__,W_ < *L*_2,N_ < *L*_2,E_. If however, both hammer throws had happened toward the same northern, eastern or western release azimuth direction β on the same sports ground (*g*), then the relation *L*_1__,N,E,W_ > *L*_2,N,E,W_ would have been remained, that is the 1. and 2. ranking numbers would not have changed (Table 7). Therefore, here mainly the effect of the Coriolis acceleration would have been responsible for the mentioned ranking number interchange.

According to Table 7, in case of the 10. (Yuriy Sedykh) and the 11. (Karl-Hans Riehm) hammer-throw world records a ranking number interchange would have happened, if both hammer-throwers had thrown at the gravitational acceleration *g*_5(7,8,9,10)_ = 9,805 m/s^2^ (of latitude φ_5_ = 0,7608 rad) with the release velocity *v* at the world records, if the 10. athlete had thrown toward west (W) and the 11. one toward east (E), when the relation *L*_10__,W_ = 80.370 m < *L*_11,E_ = 80.371 m would have been realized. (i) In this case, the Coriolis acceleration would have slightly increased the eastward (β_E_ = 90°) range of the 11. thrower, while it would have slightly decreased the westward (β_W_ = 270°) range of the 10. athlete. (ii) The smaller release velocity *v*_11_ = 27.744 m/s of the 11. athlete decreased the range relative to the 10. thrower performing larger *v*_10_ = 27.766 m/s. (iii) The smaller height *h*_10_ = 1.85 m of the 10. athlete decreased the range compared to that of the taller 11. thrower with *h*_11_ = 1.95 m (Table 3). (iv) The 10. athlete has originally thrown at smaller gravitational acceleration g_10_ = 9.805 m/s^2^, which increased his range relative to the 11. thrower, whose range was decreased by the larger g_11_ = 9.810 m/s^2^ (Table 3). Here the synergism of the release azimuth direction β, release velocity *v*, height *h* and gravitational acceleration *g* would have caused the 10. ↔ 11. ranking number interchange due to the relation *L*_10__,W_ < *L*_11,E_. If however, both hammer-throwers had thrown with the same northern, eastern or western release azimuth direction β on the same sports ground (*g*), then the relation *L*_10__,N,E,W_ > *L*_11,N,E,W_ would have remained, that is the original 10. and 11. ranking numbers would not have changed (Table 7). Here therefore, mainly the effect of the Coriolis acceleration would have been responsible for the mentioned ranking number interchange.

If the world-record rankings were based on the release velocity *v* of the hammer, then according to Table 3, the 11. ↔ 12. ranking number interchange would occur due to the relation *v*_12_ = 28.461 m/s > *v*_11_ = 28.460 m/s, furthermore, the original 18. would be the new 16., the original 16. would be the new 17., and the original 17. would be the new 18., because *v*_18_ = 27.767 m/s > *v*_16_ = 27.763 m/s > *v*_17_ = 27.759 m/s.

We have already mentioned, that the effects of different environmental circumstances of different sites of throwing events on the range could be eliminated only, if the athletes threw under the same environmental conditions. Since this is practically impossible, it would be worth considering the release velocity *v* of the shot as the ranking measure. Physically, the normalized muscle work *A* of shot-putters expressed by (11) would be even a more appropriate ranking measure. Table 8 compares the ranges *L* on the basis of *A*.

**Table 8:** Ranges *L*_i,j_ (in meter) (row: *i* = 12, 13; column: *j* = 1, 2, …, 7) of senior outdoor male shot-putters obtained with computer modelling for the release velocities *v*_i_ (m/s) calculated from (9) using the normalized muscle works *A*_i_ in Table 1, gravitational acceleration *g*_j_ (m/s^2^) at latitude φ_j_ of the throwing events, release angle α = 37°, straight *d* = 2.0 m and release azimuth angles β_N_ = 0° (north), β_E_ = 90° (east), β_W_ = 270° (west).

*H*_i_ (m)	*A*_i_ (m^2^/s^2^)	*g*_1(1,4,5,6)_ = 9.805	*g*_2(2,3,9,13,16,17,18,19,20)_ = 9.797	*g*_3(7,8)_ = 9.813	*g*_4(10)_ = 9.817	*g*_5(11)_ = 9.810	*g*_6(12)_ = 9.787	*g*_7(14,15)_ = 9.794
		φ_1_ = 0.7686	φ_2_ = 0.5945	φ_3_ = 0.9166	φ_4_ = 1.0072	φ_5_ = 0.8538	φ_6_ = 0.3721	φ_7_ = 0.5341
*i* = 12. *H*_12_ = 2.250	*i* = 12. *A*_12_ = 220.450	*k* = 12. N: 21.810	*k* = 12. N: 21.828	*k* = 12. N: 21.792	*k* = 12. N: 21.784	*k* = 12. N: 21.799	*i = 12., L*_*12*_* = 21.85*	*k* = 12. N: 21.835
							*k* = 12. N: 21.850	
		*k* = 12. E: 21.812	*k* = 12. E: 21.830	*k* = 12. E: 21.794	*k* = 12. E: 21.785	*k* = 12. E: 21.801	*k* = 12. E: 21.853	*k* = 12. E: 21.837
		***k* = 12.(13.) W: 21.808**	***k* = 12.(13.) W: 21.826**	***k* = 12.(= 13.) W: 21.791**	***k* = 12.(= 13.) W: 21.782**	***k* = 12.(= 13.) W: 21.797**	***k* = 12.(13.) W: 21.848**	***k* = 12.(13.) W: 21.832**
*i* = 13. *H*_13_ = 2.170	*i* = 13. *A*_13_ = 221.261	*k* = 13. N: 21.807	*i = 13., L*_*13*_* = 21,82*	*k* = 13. N: 21.789	*k* = 13. N: 21.780	*k* = 13. N: 21.796	*k* = 13. N: 21.847	*k* = 13. N: 21.831
			*k* = 13. N: 21.824					
		***k* = 13.(12.) E: 21.809**	***k* = 13.(12.) E: 21.827**	***k* = 13.(= 12.) E: 21.791**	***k* = 13.(= 12.) E: 21.782**	***k* = 13.(= 12.) E: 21.797**	***k* = 13.(12.) E: 21.849**	***k* = 13.(12.) E: 21.833**
		*k* = 13. W: 21.805	*k* = 13. W: 21.822	*k* = 13. W: 21.787	*k* = 13. W: 21.779	*k* = 13. W: 21.794	*k* = 13. W: 21.844	*k* = 13. W: 21.829

The italic items *L*_*12*_*, L*_*13*_ and sequential numbers *i = 12., 13.* are equal with the 12., 13. world records in Table 1. Ranges *L*_i_ in a given column *j* and the underlined sequential numbers *k* = 12., 13. represent those ranges and ranking numbers which could have been achieved in a fictive throwing event at gravitational acceleration *g*_j_, as if both athletes had thrown at the same *g*_j_ with the normalized muscle work *A*_i_ performed at the own world records with release angle α and straight *d* toward north (N: β_N_ = 0°), east (E: β_E_ = 90°) or west (W: β_W_ = 270°). The indices in parentheses of *g*_j_ are equal with the indices of the gravitational accelerations with practically the same magnitude (Δ*g*_max_ = 0.002 m/s^2^) in Table 1. In case of the bold data, *i* is not always equal with *k.*

According to Table 8, at gravitational accelerations *g*_1(1,4,5,6)_ = 9.805 m/s^2^, *g*_2(2,3,9,13,16,17,18,19,20)_ = 9.797 m/s^2^, *g*_6(12)_ = 9.787 m/s^2^ and *g*_7(14,15)_ = 9.794 m/s^2^, in case of the 12. (Terence Albritton) and 13. (Allan Feuerbach) shot-put world records the 12. ↔ 13. ranking number interchange would have occurred, if both shot puts had happened at the same *g* with the normalized muscle work *A* performed at world records, the 12. shot put toward west (W) and the 13. shot put toward east (E), when the relation *L*_12__,W_ < *L*_13,E_ would have been realized. From Table 8 it is also clear that at *g*_3(7,8)_ = 9.813 m/s^2^, *g*_4(10)_ = 9.817 m/s^2^ and *g*_5(11)_ = 9.810 m/s^2^, in case of the 12. (Terence Albritton) and 13. (Allan Feuerbach) world records, the 12. = 13. ranking number identity would have occurred, if both shot puts had happened at the same *g* with the normalized muscle work *A* achieved at world records, the 12. shot put toward west (W) and the 13. shot put toward east (E), when the relation *L*_12__,W_ = *L*_13,E_ would have been realized.

For all above cases the followings are true: (i) The Coriolis acceleration would have decreased the western (β_W_ = 270°) range of the 12. shot put, while it would have increased the eastern (β_E_ = 90°) range of the 13. shot put. (ii) The smaller normalized muscle work *A*_12_ = 220.450 m^2^/s^2^ of the 12. shot put decreased the range compared to the 13. shot put with *A*_13_ = 221.261 m^2^/s^2^. (iii) The 8 cm height advantage of the 12. athlete (*h*_12_ = 1.94 m) increased the range compared to the lower (*h*_13_ = 1.86 m) 13. thrower (Table 1). Here the synergistic effects of the release azimuth direction β, normalized muscle work *A* and height *h* would have caused the 12. ↔ 13. ranking number interchange (because *L*_12,W_ < *L*_13,E_) and the 12. = 13. ranking number identity (due to *L*_12,W_ = *L*_13,E_). However, if the shot puts had happened toward the same northern, eastern or western release azimuth direction at the same gravitational acceleration *g*, then the original relation *L*_12__,N,E,W_ > *L*_13,N,E,W_ would have remained, that is the 12. and 13. ranking numbers would not have changed (Table 8). Therefore, here mainly the effect of the Coriolis acceleration would have been responsible for the mentioned interchange or identity of these ranking numbers.

If the world-record rankings were based on the normalized muscle work *A* of shot-putters, then the 12. ↔ 13. ranking number interchange would occur due to the relation *A*_12_ = 220.450 m^2^/s^2^ < *A*_13_ = 221.261 m^2^/s^2^. Furthermore, the 19. ↔ 20. ranking number interchange would occur, because *A*_20_ = 202.934 m^2^/s^2^ > *A*_19_ = 202.826 m^2^/s^2^ (Table 1).

## Discussion

In this work, we analysed the best 20, 20, 20, 20 outdoor world records of senior male and female shot-putters and hammer-throwers. We showed that the demonstrated changes of ranking orders are related to the release height *H* (practically proportional to the athlete’s height *h*), the latitude (φ)-dependent local gravitational acceleration *g* (i.e. centrifugal acceleration) and the throwing azimuth direction β (i.e. Coriolis acceleration). The release velocities *v* of the 80 studied world records were computationally reconstructed as the physical basis of a correct comparison of throwing performances. Since the concrete values of the release angle α are unknown, in our computations we necessarily had to assume the ideal α-values (α_shot_ = 37° and α_hammer_ = 44°). Similarly, since the actual values of the air density ρ (determined by the air temperature *T* and air pressure *p*) and ground obliquity *GO* during the 80 investigated world records are unknown, in our computer simulations constant values of *T*, *p*, ρ and *GO* were necessarily assumed (*T* = 293 K, *p* = 101 325 Pa, ρ = 1.21 kg/m^3^, *GO* = 0). We revealed whether a given change of the world record ranking is caused by differences in the athlete’s height *h* and/or the local gravitational acceleration *g* (centrifugal acceleration) and/or the throwing azimuth direction β (Coriolis acceleration). It is evident that in certain cases the centrifugal and Coriolis accelerations have traceable influence upon performance.

We necessarily made a few generalities/assumptions about the air temperature *T*, air pressure *p*, air density ρ, wind velocity *v*_wind_, altitude *AL*, ground obliquity *GO*, as well as average release angle α and release height H*. Although there is an average amongst throwers, we could not take into account the environmental changes and the individual variability based on the athletes anthropometrics, technique, or attempt taken, because the actual values of *T*, *p*, ρ, *v*_wind_, *AL*, *GO*, α and H* in a given throwing event are unknown and cannot be find out.

It is well-known that on the northern/southern hemisphere, the Coriolis acceleration deviates the trajectory of an implement clockwise/counter-clockwise. This phenomenon is, of course, involved in our computer model, in the motion Eq. (13) of which the Coriolis acceleration occurs in a vectorial form. Among the 40 + 40 shot put and hammer throw world records studied by us, only the hammer throw of the Polish Anita Wlodarczyk happened on the southern hemisphere, in Rio de Janeiro (Brazil) at latitude φ = − 0.3999 rad (Table 4). The 79 other throwing events have been performed on the northern hemisphere (Tables 1, 2, 3 and 4). However, our study focused on the range and the computationally reconstructed release velocity, rather than on this clockwise/counter-clockwise deviation of the implements.

The two main methods of shot put are the rotation and glide techniques. The glide technique has decreased in its utilization by athletes and coaches over time, while the rotational technique has become more widely adopted in sport. In such time the world records have increased, in large by rotational shot-putters. Since we could not find out which technique was used in the 2 × 20 = 40 world records of shot put, in our analysis this issue was not considered.

Under given environmental conditions (latitude, release azimuth direction, altitude, ground obliquity and meteorological circumstances) and at a given release height *H* and release angle α, the range *L* is determined exclusively by the release velocity *v*. In this case, the larger the *v*, the longer is *L*. Therefore, on a given throwing event (at the same location, point of time and meteorological conditions) *v* and *L* are equally good measure for ranking records. The problem is, however, that throwing events usually happen at different locations, times and meteorological circumstances, thus *L* can vary due to the varying environmental conditions, even if *H*, α and *v* were not change. The question is whether a thrower using two different techniques (glide or rotation) can achieve the same release height *H*, release angle α and release velocity *v*. This could be investigated in the future.

We hypothesised that certain world-record ranking numbers of shot put and hammer throw may change if the effects of the athlete’s height, centrifugal and Coriolis accelerations on range were taken into account. We showed here that some ranking numbers would indeed change in this case. Here we deal only with the shot put and hammer throw in which aerodynamics plays a secondary role due to the relatively small surface area per weight ratio of the shot and hammer. In the discus and javelin throws, the effect of aerodynamics-determined environmental factors on range and its computer modelling are much complicated. This could be an interesting task of future research.

Mizera and Horváth^22^ have pointed out that if the influences of environmental factors on the range of shot put and hammer throw are not taken into account in the approval of the new world records, the championship ranking tables may incorrectly represent the real performances of shot-putters and hammer-throwers. Ranges achieved by different throwers with different release azimuth directions and at various latitudes (gravitational accelerations) cannot be correctly compared with each other. The comparison done until now is physically incorrect, because certain athletes may possess an unfair advantage/disadvantage against other athletes throwing under more disadvantageous/advantageous conditions (azimuth directions and/or latitudes). Mizera and Horváth^22^ have predicted that it is imaginable that after taking into account the influence of certain environmental factors on range, the hammer-throw and shot-put ranking numbers could change. Since the air pressure, air temperature and wind speed during earlier throwing events are practically unknown, the most one can reconstruct subsequently is the magnitude with which the centrifugal and Coriolis accelerations induced by Earth’s rotation helped or hindered the throwers.

Here we demonstrated that the world-record rankings of the shot put and hammer throw are also determined by the height *h* of athletes, because a larger/smaller *h* results in an advantage/disadvantage, since it increases/decreases the range *L*. Since the values of ratio *h*/*L* of the hammer throw are much smaller than those of the shot put due to the much smaller ranges of the latter, the influence of *h* on the rankings based on *L* is smaller in case of the hammer throw. These rankings are also affected by the gravitational acceleration *g* depending on the latitude φ, because a larger/smaller *g* means a disadvantage/advantage, since it decreases/increases *L*. This effect was only small up to the present day, because φ of the throwing events ranged in a relatively narrow interval, and thus *g* changed only slightly.

In order to eliminate the advantages/disadvantages concerning the range *L* originating from the latitude dependence of the gravitational acceleration *g*, the ranges *L*(*g*) achieved at different *g* (of different latitudes φ) should be recalculated for a given reference *g*_ref_, and the world-record rankings should be based on ranges *L*(*g*_ref_). In this case, beyond the other relevant environmental impacts (mainly the air drag depending on the air density and wind speed), the ranking ranges *L* would be determined essentially only by the release velocity *v* of the shot/hammer and how precisely are able the throwers to keep the ideal release angle α ensuring maximal *L*. Therefore, it would be more pertinent to consider the release velocity *v* of the shot/hammer as the measure of the real, own performance of throwers being almost independent of environmental factors. The world-record rankings should be based on *v*, instead of the range depending on environmental conditions. The release velocity *v* of the shot/hammer can be easily measured in situ with an equipment based on the Doppler’s effect of ultrasound or laser.

Since the release velocity *v* of the shot depends on the gravitational acceleration *g*, only the normalized muscle work *A* performed by the shot-putter – being practically independent of the environmental conditions – can be considered as the real physical performance of the shot-putter, rather than the *v* or the range more or less depending on several environmental factors. Thus, the normalized muscle work *A*, being independent of *g*, characterizes the shot-putter’s performance even more correctly than *v*. Consequently, it would be worth determining the normalized muscle work *A* of shot-putters^35^ as the most relevant ranking measure and determining the world-record rankings of the shot put on the basis of *A*.

In case of points marked by * of the undermentioned findings, the conclusions of our computer simulations were the same as those of the analytical calculations^28^.

If the release velocity *v* of the shot or the normalized muscle work *A* of the shot-putters were the ranking measure, then the following ranking number interchanges ( ↔) would occur in the male and female shot put world-record rankings:

*male shot put: 12. (Terence Albritton) ↔ 13. (Allan Feuerbach)

*male shot put: 19. (Dallas Long) ↔ 20. (Williem Nieder)

*female shot put: 3. (Natalya Lisovskaya) ↔ 4. (Ilona Slupianek)

*female shot put: 11. (Marianne Adam) ↔ 12. (Helena Fibingerova).

If the release velocity *v* of the hammer were the ranking measure, then the following interchange ( ↔) and changes ( →) of the ranking numbers would occur in the male hammer throw world-record ranking:

*male hammer throw: 11. (Karl-Hans Riehm) ↔ 12. (Boris Zaychuk)

*male hammer throw: 16. (Karl-Hans Riehm) → 17. (Aleksey Spiridonov)﻿

*male hammer throw: 17. (Aleksey Spiridonov) → 18. (Reinhard Theimer)﻿

*male hammer throw: 18. (Reinhard Theimer) → 16. (Karl-Hans Riehm)﻿

### If the throws had happened with the release velocities achieved at world records, toward different azimuths at various latitudes

*If the 19. (Nadezhda Chizhova) and the 20. (Margitta Gummel) shot-putters had thrown at the same gravitational acceleration *g* with the release velocity *v* at world records, then independently of the release azimuth direction and local *g*, the originally equal ranges *L*_19_ = *L*_20_ = 20.10 m would have changed in such a way that the range of Nadezhda Chizhova would have been smaller than the range of Margitta Gummel (*L*_19,N,E,W_ < *L*_20,N,E,W_), that is their ranking numbers would have interchanged: 19. ↔ 20.

If the 12. (Terence Albritton) shot-putter had thrown toward west (W) and the 13. (Allan Feuerbach) toward east (E) with the release velocity *v* at world records at the same *g* = 9.787 m/s^2^, then both ranges would have been the same: *L*_12,W_ = *L*_13,E_ = 21.848 m, that is the 12. = 13. ranking number identity would have occurred. If however, both shot-putters had thrown toward north, east or west at *g* = 9.787 m/s^2^, then the 12. and 13. ranking numbers would not have changed.

If the 1. (Yuriy Sedykh) hammer-thrower had thrown toward north (N) and the 2. (Yuriy Sedykh) toward east (E) at the same *g* with the release velocity *v* at world records, then independently of the local *g*, the 1. ↔ 2. ranking number interchange would have occurred because of the relation *L*_1__,N_ < *L*_2,E_.

If the 1. (Yuriy Sedykh) hammer-thrower had thrown toward west (W) and the 2. (Yuriy Sedykh) toward north (N) or east (E) at the same *g* with the release velocity *v* at world records, then independently of the local *g*, the 1. ↔ 2. ranking number interchange would have occurred due to the relation *L*_1__,W_ < *L*_2,N_ < *L*_2,E_. If however, both hammer throws had happened toward north, east or west at the same *g*, then the 1. and 2. world-record ranking numbers of the male hammer throw would not have changed.

If the 10. (Yuriy Sedykh) hammer-thrower had thrown toward west (W) and the 11. (Karl-Hans Riehm) toward east (E) with the release velocity *v* at world records at the same *g* = 9.805 m/s^2^, then the range of Yuriy Sedykh would have been smaller than the range of Karl-Hans Riehm (*L*_10,W_ < *L*_11,E_), that is their ranking numbers would have interchanged: 10. ↔ 11. If however, both hammer-throwers had thrown toward north, east or west at *g* = 9.805 m/s^2^, then the 10. and 11. ranking numbers of the male hammer throw would not have changed.

If the 12. (Mihaela Melinte) hammer-thrower had thrown toward west (W) and the 13. (Mihaela Melinte) toward east (E) with the release velocity *v* at world records at the same *g* = 9.788 m/s^2^, then both ranges would have been the same: *L*_12,W_ = *L*_13,E_ = 76.207 m, that is then the 12. = 13. ranking number identity would have occurred. However, since Mihaela Melinte performed her 12. and 13. world records on the same sports ground (*g*) and day toward the same release azimuth direction, therefore the relation *L*_12_ > *L*_13_ does not change yet, that is the 12. and 13. ranking numbers of the female hammer throw do not change.

### If the shot put had happened with the normalized muscle work achieved at world records, toward different azimuths at various latitudes

If the 12. (Terence Albritton) shot-putter had thrown toward west (W) and the 13. (Allan Feuerbach) toward east (E) at the same *g* = 9.805, 9.797, 9.787 or 9.794 m/s^2^ with the normalized muscle work *A* at world records, then due to the relation *L*_12__,W_ < *L*_13,E_ the 12. ↔ 13. ranking number interchange of the male shot put would have occurred.

If the 12. (Terence Albritton) shot-putter had thrown toward west (W) and the 13. (Allan Feuerbach) toward east (E) at the same *g* = 9.813, 9.817 or 9.810 m/s^2^ with the normalized muscle work *A* at world records, then the 12. = 13. ranking number identity would have occurred because of the relation *L*_12__,W_ = *L*_13,E_. If however, the shot puts had happened toward north, east or west release azimuth direction at the same local *g*, then the 12. and 13. world-record ranking numbers of the male shot put would not have changed.

If the 19. (Nadezhda Chizhova) and the 20. (Margitta Gummel) shot-putters had thrown at the same *g* with the normalized muscle work *A* at world records, then independently of the release azimuth direction and local *g*, the originally equal ranges *L*_19_ = *L*_20_ = 20.10 m would have changed so that the range of Nadezhda Chizhova would have been smaller than the range of Margitta Gummel (*L*_19,N,E,W_ < *L*_20,N,E,W_), that is the 19. and 20. ranking numbers of the female shot put would have interchanged.

### Comparison of the effects of different parameters

Mizera and Horváth^22^ studied in detail the influences of environmental factors on the range *L* of shot put and hammer throw. These influences are summarized in Table 9, from which one can see for the hammer throw that the maximal effect (Δ*L*_max_ = 4.5 dm) of the centrifugal acceleration (i.e. latitude φ) on *L* is (i) comparable to the maximal effect of the air temprature *T* (3.5 dm) and ground obliquity *GO* (1.74 dm), (ii) smaller than the maximal effect of the altitude *AL* (1 m) and wind speed *w* (0.7 m), and (iii) larger than the maximal effect of the air pressure *p* (9.4 cm). For hammer throw, the maximal effect (Δ*L*_max_ = 1.5–4 cm) of the Coriolis acceleration (i.e. release azimuth angle β) is smaller than the maximal effects of *GO*, *T*, *AL*, *w* and *p*.

**Table 9:** Possible maximal change Δ*L*_max_ of the range *L* because of the possible maximal change of different environmental factors computed for the male (Yuriy Sedykh) world-record hammer throw (*L* = 86.74 m) and the male (Randy Barnes) shot put (*L* = 23.12 m) (adapted and modified from Table 7 in^22^).

Variable	Change of variable (from to: →)	Environmental factor(s)	Hammer throw (*L* = 86.74 m) Δ*L*_max_	Shot put (*L* = 23.12 m) Δ*L*_max_
Latitude φ	Δφ = 90°, poles → equator, eastern throw (β = 270°)	Centrifugal acceleration	45 cm	11 cm
Release azimuthangle β	Δβ = 180°, eastern → western throw on the equator (φ = 0°)	Coriolis acceleration	4 cm	8 mm
Release azimuth angle β	Δβ = 40°, left → right margin of the throwing sector, northern or southern orientation of the throwing platform	Coriolis acceleration	1.5 cm	2 mm
Altitude *AL* = 0 m	Δ*H* = 3500 m	Gravitation, air density (air drag)	1 m	8 cm
Ground obliquity *GO* = 0	Δ*GO* = ± 1:1000	Gravitation	1.74 dm	4.6 cm
Air pressure *p* = 100 kPa	Δ*p* = ± 2 kPa	Air density (air drag)	9.4 cm	6 mm
Air temperature *T* = 20 °C	Δ*T* = ± 20 °C	Air density (air drag)	3.5 dm	2.4 cm
Wind speed *w* = 0 m/s	Δ*w* = ± 2 m/s	Air drag	0.7 m	9 cm

In the case of shot put (Table 9), the maximal effect (Δ*L*_max_ = 11 cm) of the centrifugal acceleration on *L* is (i) slightly larger than but comparable to the maximal effect of *w* (9 cm) and *AL* (8 cm), and (ii) much larger than the maximal effects of *GO* (4.6 cm), *T* (2.4 cm) and *p* (6 mm). For shot put, the maximal effect (Δ*L*_max_ = 2–8 mm) of the Coriolis acceleration is (i) much smaller than the maximal effects of *GO*, *T*, *AL* and *w*, and (ii) comparable to the maximal effect of *p*.

**Figure 5:**
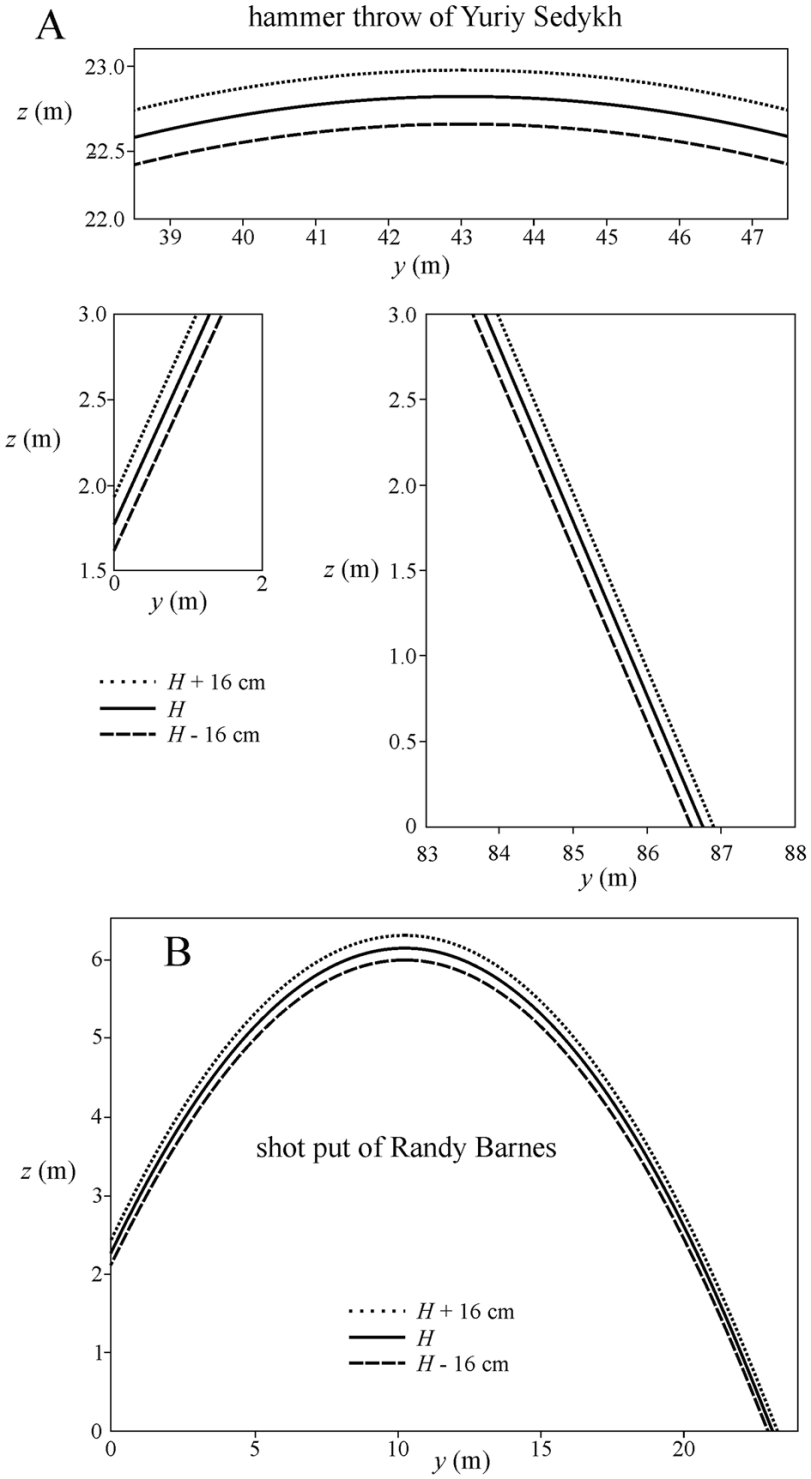
Trajectories of the hammer and shot computed under normal environmental conditions (*T*_air_ = 293 K, *p*_air_ = 101 325 Pa, ρ_air_ = 1.21 kg/m^3^, ground obliquity *GO* = 0, altitude *AL* = 0 m, release azimuth angle β = 0° = North) for the world-record hammer throw (range *L* = 86.74 m at release height *H* = 1.778 m) of Yuriy Sedykh (**A**) and the world-record shot put (*L* = 23.12 m at *H* = 2.260 m) of Randy Barnes (**B**), when the release height *H* is decreased (+ 16 cm) and increased (-16 cm) by 16 cm.

From Tables 1, 2, 3 and 4 we can see that the maximal height difference Δ*h*_max_ of the studied athletes is 16 cm (Table 1, male shot putters: 201–186 = 15 cm; Table 2, female shot putters: 188–172 = 16 cm; Table 3, male hammer throwers: 195–180 = 15 cm; Table 4, female hammer throwers: 186–170 = 16 cm). As seen in Fig. 4I–L, the range differences Δ*L*(*H*) = *L*(*H*)–*L*_world record_ increases linearly with increasing release height *H*. According to our computer modelling, Δ*H* =  ± 16 cm difference in *H* (resulted in by the maximal height difference Δ*h*_max_ =  ± 16 cm) can influence the range *L*_hammer_(*H* = 1.778 m) = 86.74 m and *L*_shot_(*H* = 2.26 m) = 23.120 m by Δ*L*_hammer_(Δ*H* =  ± 16 cm) =  ± 15 cm, and Δ*L*_shot_(Δ*H* =  ± 16 cm) =  ± 17 cm (see Fig. 5). The absolute value of the latter is larger than that of the former, because the release angle α_shot_ = 37° is smaller than α_hammer_ = 44°.

Comparing |Δ*L*_hammer_|= 15 cm with the data in Table 9, we can see that this 15 cm effect of *h* and *H* on the range *L* of hammer throw is (i) smaller than the maximal effects of the latitude φ, altitude *AL*, air temperature *T* and wind speed *w*, (ii) comparable to the maximal influence of the ground obliquity *GO*, and (iii) larger than the maximal impacts of the release azimuth angle β and air pressure *p*. On the other hand, the |Δ*L*_shot_|= 17 cm effect of *h* and *H* on the range *L* of shot put is larger than the maximal impacts of φ, β, *AL*, *GO*, *p*, *T* and *w*.

## Conclusions

1. The release velocity *v* of shot and hammer (easily measurable with an ultrasound/laser Doppler gauge) reflects the athlete’s performance much better than the range *L*, because the latter is influenced by the latitude-dependent local gravitational acceleration *g*, athlete’s height *h* and some other environmental factors, mostly the air-drag determined by the air density and wind speed. Therefore, it would be more preferable to consider the release velocity *v* of the implement as the own performance of athletes. Since *v* is practically independent of the environmental factors, it would be more correct if the ranking orders were based on it instead of the range.
2. If the release velocity *v* of the shot or the normalized muscle work of shot-putters were considered as the ranking measure of the thrower’s performance, then a few interchanges or identities of the ranking numbers would occur in the world-record rankings of the senior outdoor female and male shot put. However, if the shot puts had the same release azimuth directions at the same local gravitational accelerations, then the ranking numbers would not have changed.
3. If the release velocity *v* of the hammer were considered as the ranking measure of the thrower’s performance, then some interchanges or changes of the ranking numbers would happen in the world-record rankings of the senior outdoor male hammer throw.
4. If the throws had happened with the release velocity *v* performed at the world record at the same gravitational acceleration *g* and toward different release azimuth directions, then certain interchanges or identities of the ranking numbers would have occurred in the world-record rankings of the senior outdoor female and male shot put and hammer throw. However, if the shot-putters and hammer-throwers had the same release azimuth directions at the same local *g*, then the ranking numbers would not have changed.
5. The largest (shot put: centimeters, hammer throw: decimeters) and smallest (millimeters) influences on the range *L* has the athlete’s height *h* and the Coriolis acceleration, respectively, while the centrifugal acceleration has a medium large influence (shot put: millimeters, hammer throw: centimeters).
6. The majority of conclusions obtained with computer simulations (modelling well the reality, taking into account the air drag and the Coriolis acceleration) are the same as those based on the analytical calculations neglecting the air drag and the Coriolis effect. The reason for this is that relative to the weight of the implement, both the air drag and Coriolis forces are very small, which thus affect only slightly the range.

## Supplementary Information

Supplementary Tables.

## Data Availability

Our paper and its Electronic Supplementary Material (Supplementary Tables S1–S4) contain all relevant data used in this study.
