# Supplementary material for: Change of world-record rankings of shot put and hammer throw due to the effects of Earth rotation and athlete’s height
Source: Sci Rep. 2023 Jun 27;13:10409. doi: 10.1038/s41598-023-36665-5 (PMC10300113; doi:10.1038/s41598-023-36665-5)
Supplement: Supplementary file 1 — Supplementary Tables. [file 41598_2023_36665_MOESM1_ESM.docx]

**Supplementary Information**

**for**

**Change of world-record rankings of shot put and hammer throw due to the effects of Earth rotation and athlete’s height**

Gábor Horváth^1,*^, Dénes Hegedűs, Judit Slíz-Balogh

1: Department of Biological Physics, Physical Institute, ELTE Eötvös Loránd University,

H-1117 Budapest, Pázmány Péter sétány 1, Hungary,

*Corresponding author, e-mail: gh@arago.elte.hu (ORCID: 0000-0002-9008-2411)

This file contains the following: Supplemenatary Table S1, S2, S3, S4

**Supplemenatary Table S1**: Height *h*_i_ (m), location and date of the 20 best consecutive world recorder outdoor senior male shot-putters (https://www.worldathletics.org/records/by-progression/15758), furthermore the internet sources of these informations. *i*: ranking number.

The heights *h* of outdoor senior male and female shot-putters and hammer-throwers performing the 20 best consecutive world records originate from public internet sources (Supplemenatary Tables S1-S4). Only the height of the American shot-putter Terence Hillary Albritton was not available from such a public source. However, in March 1977 Tom Jordan has interviewed Albritton (Jordan, 1977). From this interview it turned out that the height of T. H. Albritton was *h* = 6 feets (= 1.822 m) + 4.5 inches (= 0.1143 m) = 1.94 m. Table 1 and Supplemenatary Table S1 contain this value.

| ***i*** | **thrower’s name**  **(nationality)** | ***h*_i_**  **(m)** | **location** | **date** | **source** |
| --- | --- | --- | --- | --- | --- |
| **1.** | Ryan Crouser  (USA) | 2.01 | Hayward Field,  Eugene,  OR (USA) | 2021  June 18 | https://en.wikipedia.org/wiki/Ryan_Crouser |
| **2.** | Randy Barnes  (USA) | 1.95 | Westwood,  CA (USA) | 1990  May 20 | https://en.wikipedia.org/wiki/Randy_Barnes |
| **3.** | Ulf Timmermann  (GDR) | 1.94 | Chania  (GRE) | 1988  May 22 | https://en.wikipedia.org/wiki/Ulf_Timmermann |
| **4.** | Alessandro Andrei  (ITA) | 1.91 | Viareggio  (ITA) | 1987  August 12 | https://en.wikipedia.org/wiki/Alessandro_Andrei |
| **5.** | Alessandro Andrei  (ITA) | 1.91 | Viareggio  (ITA) | 1987  August 12 | https://en.wikipedia.org/wiki/Alessandro_Andrei |
| **6.** | Alessandro Andrei  (ITA) | 1.91 | Viareggio  (ITA) | 1987  August 12 | https://en.wikipedia.org/wiki/Alessandro_Andrei |
| **7.** | Udo Beyer  (GDR) | 1.94 | Berlin  (GDR) | 1986  August 21 | https://en.wikipedia.org/wiki/Udo_Beyer |
| **8.** | Ulf Timmermann  (GDR) | 1.94 | Berlin  (GDR) | 1985  September 22 | https://en.wikipedia.org/wiki/Ulf_Timmermann |
| **9.** | Udo Beyer  (GDR) | 1.94 | Los Angeles,  CA (USA) | 1983  June 25 | https://en.wikipedia.org/wiki/Udo_Beyer |
| **10.** | Udo Beyer  (GDR) | 1.94 | Göteborg  (SWE) | 1978  July 6 | https://en.wikipedia.org/wiki/Udo_Beyer |
| **11.** | Aleksandr Baryshnikov  (URS) | 1.98 | Colombes  (FRA) | 1976  July 10 | https://en.wikipedia.org/wiki/Aleksandr_Baryshnikov |
| **12.** | Terence Albritton  (USA) | 1.94 | Honolulu,  HI (USA) | 1976  February 21 | Tom Jordan (1977) |
| **13.** | Allan Feuerbach  (USA) | 1.86 | San Jose  CA (USA) | 1973  May 5 | https://en.wikipedia.org/wiki/Al_Feuerbach |
| **14.** | Randel Matson  (USA) | 2.01 | College Station,  TX (USA) | 1967  April 22 | https://en.wikipedia.org/wiki/Randy_Matson |
| **15.** | Randel Matson  (USA) | 2.01 | College Station,  TX (USA) | 1965  May 8 | https://en.wikipedia.org/wiki/Randy_Matson |
| **16.** | Dallas Long  (USA) | 1.93 | Los Angeles,  CA (USA) | 1964  July 25 | https://en.wikipedia.org/wiki/Dallas_Long |
| **17.** | Dallas Long  (USA) | 1.93 | Los Angeles,  CA (USA) | 1964  May 29 | https://en.wikipedia.org/wiki/Dallas_Long |
| **18.** | Dallas Long  (USA) | 1.93 | Los Angeles,  CA (USA) | 1964  April 4 | https://en.wikipedia.org/wiki/Dallas_Long |
| **19.** | Dallas Long  (USA) | 1.93 | Los Angeles,  CA (USA) | 1962  May 18 | https://en.wikipedia.org/wiki/Dallas_Long |
| **20.** | William Nieder  (USA) | 1.90 | Walnut,  CA (USA) | 1960  August 12 | https://en.wikipedia.org/wiki/Bill_Nieder |

**Supplemenatary Table S2**: Height *h*_i_ (m), location and date of the 20 best consecutive world recorder outdoor senior female shot-putters (https://www.worldathletics.org/records/by-progression/5543), furthermore the internet sources of these informations. *i*: ranking number.

| ***i*** | **thrower’s name**  **(nationality)** | ***h*_i_**  **(m)** | **location** | **date** | **source** |
| --- | --- | --- | --- | --- | --- |
| **1.** | Natalya Lisovskaya  (URS) | 1.88 | Moskva  (URS) | 1987  June 7 | https://en.wikipedia.org/wiki/Natalya_Lisovskaya |
| **2.** | Natalya Lisovskaya  (URS) | 1.88 | Moskva  (URS) | 1987  June 7 | https://en.wikipedia.org/wiki/Natalya_Lisovskaya |
| **3.** | Natalya Lisovskaya  (URS) | 1.88 | Sochi  (URS) | 1984  May 27 | https://en.wikipedia.org/wiki/Natalya_Lisovskaya |
| **4.** | Ilona Slupianek  (GDR) | 1.79 | Potsdam  (GDR) | 1980  May 11 | https://en.wikipedia.org/wiki/Ilona_Slupianek |
| **5.** | Ilona Slupianek  (GDR) | 1.79 | Celje  (SLO) | 1980  May 2 | https://en.wikipedia.org/wiki/Ilona_Slupianek |
| **6.** | Helena Fibingerova  (TCH) | 1.79 | Nitra  (TCH) | 1977  August 20 | https://en.wikipedia.org/wiki/Helena_Fibingerov%C3%A1 |
| **7.** | Helena Fibingerova  (TCH) | 1.79 | Opava  (TCH) | 1976  September 26 | https://en.wikipedia.org/wiki/Helena_Fibingerov%C3%A1 |
| **8.** | Ivanka Khristova  (BUL) | 1.72 | Belmeken  (BUL) | 1976  July 4 | https://en.wikipedia.org/wiki/Ivanka_Khristova |
| **9.** | Ivanka Khristova  (BUL) | 1.72 | Belmeken  (BUL) | 1976  July 3 | https://en.wikipedia.org/wiki/Ivanka_Khristova |
| **10.** | Marianne Adam  (GDR) | 1.83 | Karl Marx Stadt  - Chemnitz  (GDR) | 1976  May 30 | https://en.wikipedia.org/wiki/Marianne_Adam |
| **11.** | Marianne Adam  (GDR) | 1.83 | Berlin  (GDR) | 1975  August 6 | https://en.wikipedia.org/wiki/Marianne_Adam |
| **12.** | Helena Fibingerova  (TCH) | 1.79 | Gottwaldov  - Zlin (TCH) | 1974  September 21 | https://en.wikipedia.org/wiki/Helena_Fibingerov%C3%A1 |
| **13.** | Nadezhda Chizhova  (URS) | 1.74 | Varna  (BUL) | 1973  September 29 | https://en.wikipedia.org/wiki/Nadezhda_Chizhova |
| **14.** | Nadezhda Chizhova  (URS) | 1.74 | Lvov  (URS) | 1973  August 28 | https://en.wikipedia.org/wiki/Nadezhda_Chizhova |
| **15.** | Nadezhda Chizhova  (URS) | 1.74 | München  (GER) | 1972  September 7 | https://en.wikipedia.org/wiki/Nadezhda_Chizhova |
| **16.** | Nadezhda Chizhova  (URS) | 1.74 | Sochi  (URS) | 1972  May 19 | https://en.wikipedia.org/wiki/Nadezhda_Chizhova |
| **17.** | Nadezhda Chizhova  (URS) | 1.74 | Moskva  (URS) | 1971  August 29 | https://en.wikipedia.org/wiki/Nadezhda_Chizhova |
| **18.** | Nadezhda Chizhova (URS) | 1.74 | Athina  (GRE) | 1969  September 16 | https://en.wikipedia.org/wiki/Nadezhda_Chizhova |
| **19.** | Nadezhda Chizhova  (URS) | 1.74 | Athina  (GRE) | 1969  September 16 | https://en.wikipedia.org/wiki/Nadezhda_Chizhova |
| **20.** | Margitta Gummel  (GDR) | 1.77 | Berlin  (GDR) | 1969  September 11 | https://en.wikipedia.org/wiki/Margitta_Gummel |

**Supplemenatary Table S3**: Height *h*_i_ (m), location and date of the 20 best consecutive world recorder outdoor senior male hammer-throwers (https://www.worldathletics.org/records/by-progression/16118), furthermore the internet sources of these informations. *i*: ranking number.

| ***i*** | **thrower’s name**  **(nationality)** | ***h*_i_**  **(m)** | **location** | **date** | **source** |
| --- | --- | --- | --- | --- | --- |
| **1.** | Yuriy Sedykh  (URS) | 1.85 | Stuttgart  (GER) | 1986  August 30 | https://en.wikipedia.org/wiki/Yuriy_Sedykh |
| **2.** | Yuriy Sedykh  (URS) | 1.85 | Tallinn  (EST) | 1986  June 22 | https://en.wikipedia.org/wiki/Yuriy_Sedykh |
| **3.** | Yuriy Sedykh  (URS) | 1.85 | Cork  (IRL) | 1984  July 3 | https://en.wikipedia.org/wiki/Yuriy_Sedykh |
| **4.** | Sergey Litvinov  (URS) | 1.80 | Moskva  (URS) | 1983  June 21 | https://en.wikipedia.org/wiki/Sergey_Litvinov_  (athlete,_born_1958) |
| **5.** | Sergey Litvinov  (URS) | 1.80 | Moskva  (URS) | 1982  June 4 | https://en.wikipedia.org/wiki/Sergey_Litvinov_  (athlete,_born_1958) |
| **6.** | Yuriy Sedykh  (URS) | 1.85 | Moskva  (URS) | 1980  July 31 | https://en.wikipedia.org/wiki/Yuriy_Sedykh |
| **7.** | Sergey Litvinov  (URS) | 1.80 | Sochi  (URS) | 1980  May 24 | https://en.wikipedia.org/wiki/Sergey_Litvinov_  (athlete,_born_1958) |
| **8.** | Yuriy Sedykh  (URS) | 1.85 | Leselidze  (URS) | 1980  May 16 | https://en.wikipedia.org/wiki/Yuriy_Sedykh |
| **9.** | Jüri Tamm  (URS) | 1.91 | Leselidze  (URS) | 1980  May 16 | https://en.wikipedia.org/wiki/J%C3%BCri_Tamm |
| **10.** | Yuriy Sedykh  (URS) | 1.85 | Leselidze  (URS) | 1980  May 16 | https://en.wikipedia.org/wiki/Yuriy_Sedykh |
| **11.** | Karl-Hans Riehm  (FRG) | 1.95 | Heidenheim  (GER) | 1978  August 6 | https://en.wikipedia.org/wiki/Karl-Hans_Riehm |
| **12.** | Boris Zaychuk  (URS) | 1.80 | Moskva  (URS) | 1978  July 9 | https://nl.wikipedia.org/wiki/Boris_Zajtsjoek |
| **13.** | Walter Schmidt  (FRG) | 1.92 | Frankfurt  am Main  (GER) | 1975  August 14 | https://www.google.com/search?client=firefox-b-d&q=height+Walter+Schmidt+hammer+throw |
| **14.** | Karl-Hans Riehm  (FRG) | 1.95 | Rehlingen  (GER) | 1975  May 19 | https://en.wikipedia.org/wiki/Karl-Hans_Riehm |
| **15.** | Karl-Hans Riehm  (FRG) | 1.95 | Rehlingen  (GER) | 1975  May 19 | https://en.wikipedia.org/wiki/Karl-Hans_Riehm |
| **16.** | Karl-Hans Riehm  (FRG) | 1.95 | Rehlingen  (GER) | 1975  May 19 | https://en.wikipedia.org/wiki/Karl-Hans_Riehm |
| **17.** | Aleksey Spiridonov  (URS) | 1.92 | München  (GER) | 1974  September 11 | https://en.wikipedia.org/wiki/Aleksey_Spiridonov |
| **18.** | Reinhard Theimer  (GDR) | 1.84 | Leipzig  (GER) | 1974  July 4 | https://en.wikipedia.org/wiki/Reinhard_Theimer |
| **19.** | Walter Schmidt  (FRG) | 1.92 | Lahr  (FRG) | 1971  September 4 | https://www.google.com/search?client=firefox-b-d&q=height+Walter+Schmidt+hammer+throw |
| **20.** | Anatoliy Bondarchuk  (URS) | 1.83 | Rovno  (UKR) | 1969  October 13 | https://en.wikipedia.org/wiki/Anatolij_  Bondar%C4%8Duk |

**Supplemenatary Table S4**: Height *h*_i_ (m), location and date of the 20 best consecutive world recorder outdoor senior female hammer-throwers (https://www.worldathletics.org/records/by-progression/5911), furthermore the internet sources of these informations. *i*: ranking number.

| ***i*** | **thrower’s name**  **(nationality)** | ***h*_i_**  **(m)** | **location** | **date** | **source** |
| --- | --- | --- | --- | --- | --- |
| **1.** | Anita Wlodarczyk  (POL) | 1.78 | Warszawa  (POL) | 2016  August 28 | https://en.wikipedia.org/wiki/Anita_W%C5%82odarczyk |
| **2.** | Anita Wlodarczyk  (POL) | 1.78 | Rio de Janeiro  (BRA) | 2016  August 15 | https://en.wikipedia.org/wiki/Anita_W%C5%82odarczyk |
| **3.** | Anita Wlodarczyk  (POL) | 1.78 | Wladyslawowo  (POL) | 2015  August 1 | https://en.wikipedia.org/wiki/Anita_W%C5%82odarczyk |
| **4.** | Anita Wlodarczyk  (POL) | 1.78 | Berlin  (GER) | 2014  August 31 | https://en.wikipedia.org/wiki/Anita_W%C5%82odarczyk |
| **5.** | Betty Heidler  (GER) | 1.75 | Halle  (GER) | 2011  May 21 | https://en.wikipedia.org/wiki/Betty_Heidler |
| **6.** | Anita Wlodarczyk  (POL) | 1.78 | Bydgoszcz  (POL) | 2010  June 6 | https://en.wikipedia.org/wiki/Anita_W%C5%82odarczyk |
| **7.** | Anita Wlodarczyk  (POL) | 1.78 | Berlin  (GER) | 2009  August 22 | https://en.wikipedia.org/wiki/Anita_W%C5%82odarczyk |
| **8.** | Tatyana Lysenko  (RUS) | 1.86 | Tallinn  (EST) | 2006  August 15 | https://en.wikipedia.org/wiki/Tatyana_Lysenko |
| **9.** | Tatyana Lysenko  (RUS) | 1.86 | Zhukovskiy  (RUS) | 2006  June 24 | https://en.wikipedia.org/wiki/Tatyana_Lysenko |
| **10.** | Gulfiya Khanafeyeva  (RUS) | 1.73 | Tula  (RUS) | 2006  June 12 | https://en.wikipedia.org/wiki/Gulfiya_Khanafeyeva |
| **11.** | Tatyana Lysenko  (RUS) | 1.86 | Moskva  (RUS) | 2005  July 15 | https://en.wikipedia.org/wiki/Tatyana_Lysenko |
| **12.** | Mihaela Melinte  (ROU) | 1.70 | Rüdlingen  (SUI) | 1999  August 29 | https://en.wikipedia.org/wiki/Mihaela_Melinte |
| **13.** | Mihaela Melinte  (ROU) | 1.70 | Rüdlingen  (SUI) | 1999  August 29 | https://en.wikipedia.org/wiki/Mihaela_Melinte |
| **14.** | Mihaela Melinte  (ROU) | 1.70 | Clermont-Ferrand  (FRA) | 1999  May 13 | https://en.wikipedia.org/wiki/Mihaela_Melinte |
| **15.** | Mihaela Melinte  (ROU) | 1.70 | Clermont-Ferrand  (FRA) | 1999  May 13 | https://en.wikipedia.org/wiki/Mihaela_Melinte |
| **16.** | Mihaela Melinte  (ROU) | 1.70 | Poiana Brasov  (ROU) | 1998  July 16 | https://en.wikipedia.org/wiki/Mihaela_Melinte |
| **17.** | Olga Kuzenkova  (RUS) | 1.76 | München  (GER) | 1997  June 22 | https://en.wikipedia.org/wiki/Olga_Kuzenkova |
| **18.** | Olga Kuzenkova  (RUS) | 1.76 | München  (GER) | 1997  June 22 | https://en.wikipedia.org/wiki/Olga_Kuzenkova |
| **19.** | Mihaela Melinte  (ROU) | 1.70 | Bucuresti  (ROU) | 1997  March 8 | https://en.wikipedia.org/wiki/Mihaela_Melinte |
| **20.** | Mihaela Melinte  (ROU) | 1.70 | Cluj-Napoca  (ROU) | 1996  May 12 | https://en.wikipedia.org/wiki/Mihaela_Melinte |
